# Singapore’s Total Diet Study (2021–2023): Study Design, Methodology, and Relevance to Ensuring Food Safety

**DOI:** 10.3390/foods13040511

**Published:** 2024-02-06

**Authors:** Geraldine Songlen Lim, Jun Cheng Er, Kalpana Bhaskaran, Paul Sin, Ping Shen, Kah Meng Lee, Guat Shing Teo, Joachim Mun Choy Chua, Peggy Chui Fong Chew, Wei Min Ang, Joanna Lee, Sheena Wee, Yuansheng Wu, Angela Li, Joanne Sheot Harn Chan, Kyaw Thu Aung

**Affiliations:** 1 National Centre for Food Science, Singapore Food Agency, 7 International Business Park, Singapore 609919, Singaporeteo_guat_shing@sfa.gov.sg (G.S.T.); peggy_chew@sfa.gov.sg (P.C.F.C.); lee_joanna@sfa.gov.sg (J.L.); aung_kyaw_thu@sfa.gov.sg (K.T.A.); 2 School of Applied Science, Temasek Polytechnic, 21 Tampines Ave 1, Singapore 529757, Singaporepaul_sin@tp.edu.sg (P.S.); 3 Department of Food Science & Technology, National University of Singapore, Science Drive 2, Singapore 117542, Singapore; 4 School of Biological Sciences, Nanyang Technological University, 60 Nanyang Dr, Singapore 637551, Singapore

**Keywords:** total diet study, food safety, dietary exposure assessment, 24 h recall, food consumption, cooking methods

## Abstract

A total diet study is often used to evaluate a population’s baseline dietary exposure to chemical hazards from across the diet. In 2021–2023, Singapore carried out a TDS, and this article presents an overview of the study design and methodological selections in Singapore’s TDS, as well as its relevance to ensuring food safety. A food consumption survey was conducted on Singapore citizens and permanent residents, where food consumption patterns of the Singapore population were identified. The selection of chemical hazards and foods for inclusion in Singapore’s TDS, as well as principal considerations on sampling, food preparation, and analytical testing are discussed. Commonly consumed foods by the Singapore population in food categories such as grain and grain-based products, meat and meat products, fish and seafood, vegetables, fruits, milk and dairy products were included in this study, and mean concentrations of chemicals tested in each food category were reported, with food categories possessing higher levels identified. Future work will include dietary exposure assessments for the population and analysis of the contributions by food and cooking method.

## 1. Introduction

A total diet study (TDS) is often applied as a tool to evaluate the population’s dietary exposures to chemical hazards from across the diet and to assess their potential impact on human health. The World Health Organization (WHO) recommends TDS as the most cost-effective method to evaluate a population’s dietary exposure to chemicals [1] and in 2011, a guidance document was published together with the Food and Agriculture Organization of the United Nations (FAO) and the European Food Safety Authority (EFSA) to harmonise the approach in conducting a TDS across countries to improve comparability [2]. Some unique features of TDS include (i) the selection of foods that are commonly consumed by the population of interest, (ii) the pooling and homogenising of foods, and (iii) foods that are analysed in a state that is ready for consumption [2].

TDSs have been conducted by many countries arising from the multitude of benefits and uses it brings to ensure food safety. In tandem with surveillance monitoring programmes, a TDS augments a country’s food safety system as both programmes differ in purpose and scope. One key difference with surveillance monitoring programmes is that a TDS encompasses a more downstream coverage of the farm-to-fork chain as samples submitted for testing are in a consumption-ready state, where foods have undergone preparation, such as washing, removal of inedible parts, and cooking, and serves as a closer representation of foods at the point of consumption. Other benefits of a TDS include its ability to provide information on the contributions of different food categories, foods, and cooking methods to the overall dietary exposure to chemical hazards. Chemical hazards that require immediate actions to be taken or close monitoring can be identified, allowing countries to prioritise resources within a given time frame. A TDS provides the baseline levels of chemicals in foods that can serve as a reference for comparison, allowing early and quick identification of any deviation or irregularity. In addition, a TDS, when conducted over many years, allows for the monitoring of trends on contaminant levels and a population’s dietary exposures. The success of measures adopted in lowering the population’s dietary exposures can also be monitored. For example, the population’s dietary exposures to lead had reduced approximately 7-fold over 10 years in the Australian TDS, reflecting the success of the risk management strategies adopted [3].

In today’s context, developments in food production techniques, the exploration of new food sources, climate change, as well as consumers’ increased emphasis on foods that are healthier/more nutritious/environmentally sustainable are some factors that fuel the change in consumption habits and preferences [4]. The ever-changing landscape highlights the importance for a TDS to be carried out regularly, as these factors have an impact on food safety, and consequently, policies, standards, and regulations would also need to be refined in order to keep abreast of these changes. 

The calculation of a population’s dietary exposure requires food consumption data. To obtain an accurate assessment of the population’s dietary exposure, the use of food consumption data reflective of the population’s consumption is preferred. A characteristic trait of the Singapore population is its multi-racial nature, largely comprising Chinese, Malays, Indians, and Others. This diverse mix results in a multi-cultural society that exerts an influence on the culinary exposure and diet of the population. While individuals of each race possess their own consumption preferences and may largely consume foods that are ethnic-specific [5], the co-existence of different cuisines in Singapore has heavily influenced the country’s culinary landscape, rendering it commonplace to observe the cross-consumption of foods by individuals from a different race [6]. The hawker culture [7,8] is also an integral part of Singapore and the abundance of both traditional ethnic and international cuisines [5] coupled with the diversity of the population and widespread consumption of foods across ethnic groups makes this a distinctive feature of the Singapore population. 

While the TDS guidance document serves as a reference in the design of a country’s TDS, there are still aspects that may vary across countries, as these are dependent on the populations’ preference, and resources available. For instance, the level of pooling, whether condiments such as salt and oil are added during food preparation, and the cooking methods adopted for each food are important features that should be understood before comparisons across countries are made. 

A TDS was carried out in 2021–2023 based on foods that are commonly consumed by the Singapore population, to obtain estimates of the current average dietary exposure to chemical contaminants by our local population, and the main foods that contribute to this exposure. Prior to this, a food consumption survey was conducted on Singapore citizens and permanent residents, where commonly consumed foods along with the average consumption amounts were obtained. Food categories analysed in this study include but are not limited to grain and grain-based products, meat and meat products, fish and seafood, vegetables, fruits, milk and dairy products. Findings from this study would ultimately be useful to enhance food safety risk management and communications based on the local diet and food supply and in the identification of specific foods for further review to enhance food safety. This paper presents an overview of the study design and methodological selections of Singapore’s TDS, and its relevance to ensuring food safety. 

## 2. Materials and Methods

This section presents the key steps in Singapore’s TDS, which involves (i) a review of chemical hazards for inclusion in Singapore’s TDS, (ii) food consumption survey as a basis for the selection of foods for inclusion in this study, and (iii) processes from sampling to analytical testing, including food preparation, pooling, storage, and transportation of samples.

### 2.1. Review of Chemical Hazards for Inclusion in Singapore’s TDS

Chemicals in foods can arise from natural sources, processing, or be added intentionally along the food supply chain. Consequently, many chemicals can possibly exist in foods; hence, guidance from international reports and meetings was taken to inform our selection on the chemicals for inclusion in Singapore’s TDS.

#### 2.1.1. Chemical Hazards from JECFA and JMPR Reports

JECFA (Joint FAO/WHO Expert Committee on Food Additives) and JMPR (Joint FAO/WHO Meeting on Pesticide Residues) reports from the preceding 20 years (since 2001) at the time of planning the study in 2021 were used in the compilation of chemical hazards to be included in Singapore’s TDS. JECFA and JMPR are independent scientific expert committees that perform risk assessments on chemicals such as food additives, contaminants, veterinary drugs, and pesticide residues. The JECFA and JMPR reports can be accessed through the following URLs, https://www.who.int/groups/joint-fao-who-expert-committee-on-food-additives-(jecfa)/publications/reports (accessed on 21 June 2023) and https://www.who.int/groups/joint-fao-who-meeting-on-pesticide-residues-(jmpr)/publications/reports (accessed on 21 June 2023) respectively [9,10]. Contaminants, veterinary drugs, and pesticide residues from the reports were considered in the compilation. 

#### 2.1.2. Chemicals Recommended for Inclusion at the International TDS Workshop

In addition to the chemicals compiled from the JECFA and JMPR reports, the priority list of chemicals established at the 5th international workshop on Total Diet Studies in 2015 [11,12] was also considered for inclusion. Three lists were made available—core, intermediate, and comprehensive—that countries could take into consideration for inclusion in their TDS, depending on resources available.

#### 2.1.3. Selection of Chemicals to Be Included in Singapore’s TDS

After the compilation of chemical hazards through the screening described above, a list of chemicals was prioritised with emphasis placed on chemicals (i) where preliminary risk assessment indicates a potential health risk, (ii) that can lead to severe adverse effects at likely exposure levels, (iii) that are widely occurring across multiple foods, (iv) subjected to an international convention, and (v) that can be co-analysed, requiring little or no additional cost. 

### 2.2. Food Consumption Survey as a Basis for the Selection of Foods for Inclusion in Singapore’s TDS

#### 2.2.1. Conducting 24 h Recall Interviews to Obtain Data on the Population’s Food Consumption

In Singapore’s TDS, the 24 h recall method was used to obtain food consumption data representative of the population in Singapore. In total, 2000 participants aged 15 and above were recruited for the 24 h recall survey. The sample size was estimated based on the Singapore resident population in 2020 [13], with representations from different age groups and races reflective of the population breakdown. A three-step stratified random sampling was adopted for the selection of respondents for the survey as previously described [14]. Residents including Singapore citizens and permanent residents with representation from both public and private housing types across different regions in Singapore were included in the survey. An external market research company with prior experience in conducting food consumption surveys was engaged. Participants were interviewed face-to-face between August 2021 and January 2022, and the average time needed to complete one interview was 30 min. The interviews were conducted on two non-consecutive days, covering both a weekday and weekend. Festive seasons were avoided as consumption habits during festive occasions are known to deviate from the norm [15,16], both in terms of the type and amount of food consumed, and hence may not accurately reflect the usual consumption of the population.

The survey questionnaire was designed in the four main languages of Singapore (English, Chinese, Malay, and Tamil), to facilitate the participation of residents that are unable to speak English. The survey questionnaire included questions on socio-demographics, followed by the type and amount of food and beverages consumed in the past 24 h. Other information such as brand, food preparation methods, occasion (e.g., lunch, tea break), and place of consumption (dining in or out) were also collected. During the survey, a food atlas, i.e., a collection of photographs of foods in a range of serving sizes was used to assist participants in improving the accuracy of the amounts of food and beverages reported to be consumed. iPads were used in the administration of the survey to minimise transcription errors. 

#### 2.2.2. Ethical Considerations

This study was approved by the National Centre for Food Science’s Project Review Committee (Project ID RAP21.1) of the Singapore Food Agency. Verbal informed consent was obtained from all participants in the study. Individual responses were anonymised through the use of an identifier to maintain the anonymity of all participants. 

#### 2.2.3. Recipes of Commonly Consumed Dishes in Singapore for the Analysis of Food Consumption Data

Recipes were used for the breakdown of dishes into food components/ingredients and their proportions where dishes, instead of foods, were reported in the survey. Consultants from Temasek Polytechnic (TP), who are chefs with professional culinary experience, were engaged to provide recipes that are representative of the Singapore population’s culinary preference.

The recipes were obtained from cookbooks [17,18,19,20,21] written by experienced local chefs, culinary experts or food enthusiasts, or online commercial recipes written/edited by professionals, as well as online user-generated recipes. The recipes were shortlisted based on the following elements: (1) the name of the recipe/dish, (2) a photograph of the dish, (3) list of ingredients used accompanied by information about quantity or serving size, (4) information about minor ingredients like spices, herbs, (5) method of preparation, and (6) yield. The name of the recipe/dish served to provide a quick reference to the dish and the method of preparation was used to evaluate if it was a typical Singaporean way of cooking the dish. In addition, the quantity and proportion of various ingredients used in the recipes were considered, and cross-checked against the yield. Considering all these factors, the team of experienced chefs and food technologists made an informed decision in choosing the most appropriate recipe for food preparation. The rating of the recipes proposed by online users was considered as well.

#### 2.2.4. Selection of Cooking Methods That Are Commonly Adopted

Cooking methods for foods that require cooking were determined through (i) consultation with Temasek Polytechnic (TP), and (ii) analysis of food consumption data collected from 24 h recall interviews. Cooking methods proposed by TP were selected through the consultants’ experience in the local F and B industry, interactions and exchanges with F and B providers, perusal of local cookbooks/food-related magazines, websites, blogs, social media, visits to local trade shows and F and B providers, as well as interest in advertisements, promotions, and menus from F and B providers. Foods prepared at social gatherings hosted by family, friends, and colleagues also contributed to their knowledge base of the common cooking methods applied to different foods. These avenues led to the consultants’ cumulative understanding of customers’ preferences of cooking methods for foods consumed locally. The questionnaire used in the 24 h recall interviews included questions relating to cooking method and food preparation, and collected data were used to supplement the advice provided by consultants.

### 2.3. Processes from Sampling to Analytical Testing, including but Not Limited to Food Preparation, Pooling, Storage, and Transportation of Samples

#### 2.3.1. Sampling and Purchasing of Foods

Sampling was carried out from January 2022 to July 2022, and a sampling plan was designed to ensure that representative samples were purchased during the sampling period. Fifteen sub-samples varying in brand, variety, country of origin or purchase location were purchased for each item in the TDS food list. Increased amounts were purchased for foods that (i) were cooked in multiple ways, (ii) shrink or lose water upon cooking, and (iii) possess small edible portions after the removal of inedible parts such as the shell, husk, peel, and bone. Samples were purchased from physical trips to wet markets, super- and hypermarkets, as well as through e-commerce platforms. A wet market is a marketplace where fresh meat, fish, and vegetables are sold. The term ‘wet’ is used because the floors are typically wet, arising from the melted ice used by some stalls to maintain the freshness of the produce, and also because some stalls spray water to clean their stalls. There is typically a wet and dry section in a wet market, and the wet section offers fresh produce such as meat and fish, while the dry section offers dried goods such as spices, rice, and beans, etc. [22]. Some samples such as fruits, bakery products, and ready-to-eat (RTE) savouries (e.g., crackers and chips) were also purchased from specialty shops. Popular brands and variants were included as much as possible to mimic consumer preferences, and this involved having sampling officers ask retail store staff about the popular variants, considering items placed on shelves at eye level, and using the “sort by popularity” filter for online shopping to ensure that the best-selling items were included. Foods with different import sources through the year were sampled twice during the sampling period to capture different variants and ensure the collection of a representative sample. Sampling records containing information on sub-samples such as brand, variety, weight, expiry date, and purchase location were kept.

#### 2.3.2. Food Preparation

The SFA partnered with TP for Singapore’s TDS and chefs with culinary experience were engaged for the preparation and cooking of foods. The preparation and cooking of foods were carried out in the TP kitchen. Common household practices in relation to food preparation, which include the removal of inedible parts, washing/cleaning of foods, and cutting, were adopted prior to cooking. Cooking oil was used in the cooking of TDS samples, and tap water from the Temasek Polytechnic kitchen was also used in food preparation and cooking. Both the tap water and cooking oil were collected and submitted for analytical testing. Stainless-steel utensils, cookware, and equipment were used in the food preparation and cooking.

Multiple cooking methods were used in the TDS, and definitions of each food preparation term and cooking method were standardised to eliminate ambiguity (Table S1 of Supplementary Information). Separately, records on food preparation and cooking performed on each food were also kept, as the cooking duration and treatment of each food may have differed.

#### 2.3.3. Pooling of Samples

Each food comprised fifteen sub-samples that vary in brand, variety, and country of origin or purchase location, and they were combined into a pooled sample prior to homogenisation. Sample pooling was performed at an individual food level, and foods that were cooked in multiple ways, i.e., with more than one food-cooking method combination, were pooled separately. Pooling was carried out either before or after food preparation/cooking, depending on the nature of the food. Foods that occur in uncountable units, such as grains, powder, liquid, and semi-liquid foods, were pooled prior to food preparation/cooking, while foods that occur in countable, large discrete units were cooked first before pooling to enable equal representation of each variant in the pooled food sample. For instance, rice samples were pooled prior to food preparation and mixed uniformly before washing and cooking. On the other hand, each mussel sub-sample went through food preparation and cooking first before the meat from all sub-samples was combined. Pooled samples were mixed homogeneously using blenders before smaller amounts were portioned out and submitted for analytical testing.

A total of 281 foods were sampled in total, giving rise to 4215 samples purchased, and 6000 samples that were prepared and cooked. This eventually resulted in 494 pooled samples that were submitted for analytical testing. Foods that had different import sources through the year were purchased twice during the sampling period, once at the start (January–February) and once at the end (June to July).

#### 2.3.4. Storage and Transportation

Purchased samples were stored under appropriate conditions; perishable foods in a chiller (0 °C to 4 °C) or freezer (−20 °C to −18 °C), and shelf-stable foods in a cool, dry place. Perishable foods stored in a chiller were prepared within 72 h, while shelf-stable foods and samples stored in the freezer were prepared within one week of purchase. Samples were either transported in a designated delivery vehicle or private hire vehicles, and cold chain was maintained for perishable food samples using ice packs, dry ice, and cooler bags, while shelf-stable foods were kept cool and dry.

Food samples were stored in containers or bags made of polyethylene (PE), polypropylene (PP), or stainless-steel material, suitable for freezing and without leaching of plasticisers and bisphenol. Ziploc bags made of PE or PP material were also used. The use of PVC, PET, polycarbonate, polystyrene, or polyethersulphone (and other bisphenol derived polymers) was avoided to prevent potential leaching onto the food samples.

#### 2.3.5. Analytical Testing of Samples

Analytical testing of TDS samples was performed in-house by the National Centre of Food Science, the national food safety testing laboratory accredited under the Singapore Accreditation Council Singapore Laboratory Accreditation (SAC-Singlas) Scheme in Singapore. Quality management systems in accordance with ISO/IEC 17025 standards [23] were used, and analytical methods were fully validated prior to use. The analytical methods and limits of quantification (LOQs) used for Singapore’s TDS were developed in accordance with the guidance document [2] by WHO, FAO, and EFSA, and by taking reference from the testing methods used in other countries’ TDS [24,25,26,27,28,29,30], as well as expected levels in foods [31,32,33]. The limits of detection (LODs) and quantification (LOQs) for the test methods used in this study, as well as the method of determination are provided in Table S2 of Supplementary Information. The method validation parameters can be found in Table S3 of Supplementary Information.

#### 2.3.6. Data Analysis

Foods reported to be consumed from the food consumption survey were categorised into 22 food categories, adapted from Codex’s GSFA food category system [34]. Descriptive analysis of the consumption of food from each food category among the consumers was performed, stratified by age, gender, race, and housing type. Concentration data for the chemicals tested in foods were analysed by food categories, and the mean concentrations were reported. Left-censored data were not included in the computation of mean values.

## 3. Results

### 3.1. Chemicals Included in Singapore’s TDS

The priority list of chemicals from the TDS workshop and chemical hazards from past years’ JECFA and JMPR reports, combined with a selection process using a set of criteria, led to the list of chemical hazards included in Singapore’s TDS as shown in Table 1. The final list comprised [2] contaminants from the environment (heavy metals, POPs), chemical residues or substances intentionally added along the food production chain (pesticide residues, veterinary drug residues), naturally occurring contaminants (mycotoxins, phytotoxins), contaminants formed during food processing (processed contaminants), as well as contaminants transferred from food packaging or food contact materials.

### 3.2. Findings from the Food Consumption Survey

#### 3.2.1. Demographics of Survey Respondents

Survey responses from a total of 2014 individuals were received. Respondent demographics are shown in Table 2 and Table 3, alongside a comparison of the actual resident demographics of Singapore in 2020.

Age and race are factors that can significantly influence food consumption patterns and habits [6,35]; hence, the food consumption of these population sub-groups will be analysed in the next section.

#### 3.2.2. Food Consumption Patterns of the Singapore Population

**Figure 1:**
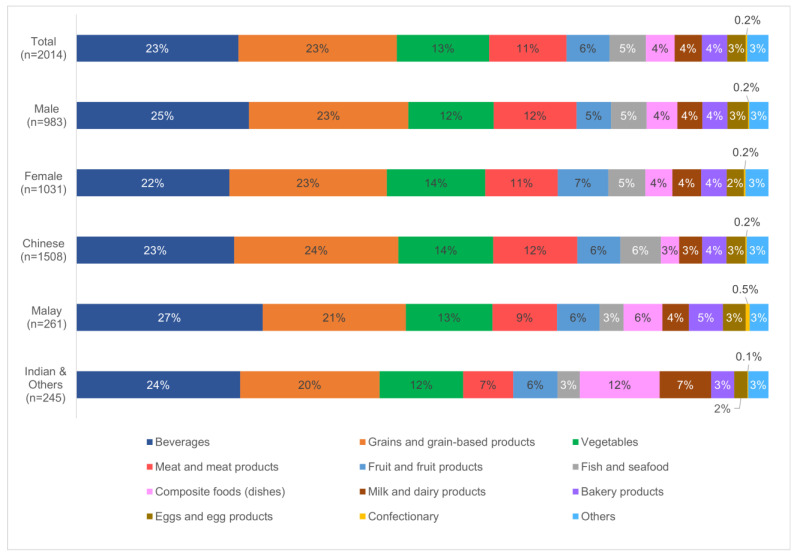
Breakdown of contributing food categories to the diet of an average Singapore adult, stratified by gender and race.

Based on data collected from two non-consecutive 24 h recall interviews, the average food consumption over two days for each respondent was taken. Figure 1 shows the breakdown of contributing food categories (excluding water) to the diet of an average Singapore adult, stratified by gender and race. Foods in each food category are provided in Table S11 of Supplementary Information.

Almost half of the average adult’s consumption in a day was attributed to beverages, and grain and grain-based products. A comparison of food consumption patterns between males and females revealed higher consumption of beverages (Male 25%, Female 22%) but lower consumption of vegetables (Female 14%, Male 12%) and fruits (Female 7%, Male 5%) among males. Among the major races in Singapore, Malays consumed the most beverages (Malay 27%, Indian and Others 24%, Chinese 23%), while the Chinese consumed more grains (Chinese 24%, Malay 21%, Indian and Others 20%), meat (Chinese 12%, Malay 9%, Indian and Others 7%), fish and seafood (Chinese 6%, Malay 3%, Indian and Others 3%). For Indians and Others, there was a notably higher consumption of composite foods (Indian and Others 12%, Malay 6%, Chinese 3%), as well as milk and dairy products (Indian and Others 7%, Malay 4%, Chinese 3%).

**Figure 2:**
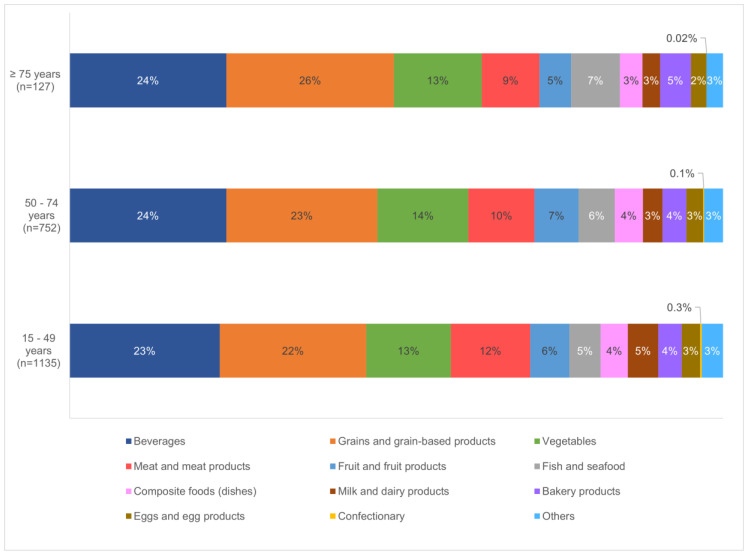
Breakdown of contributing food categories to the diet of an average Singapore adult, by age groups.

A comparison of food consumption patterns across the different age groups (Figure 2) showed similar trends in food categories that were most consumed, but a higher consumption of grain and grain-based products was observed among individuals aged 75 and above (≥75 years 26%, 50–74 years 23%, 15–49 years 22%). On the other hand, those aged between 15 and 49 years consumed more meat (15–49 years 12%, 50–74 years 10%, ≥75 years 9%), and milk and dairy products (15–49 years 5%, 50–74 years 3%, ≥75 years 3%). It was also observed that while confectionary is lesser consumed by the population, individuals aged 75 and above almost do not consume them based on the survey. The foods and beverages consumed by the population, together with the consumption amounts, can be found in Table S4 of Supplementary Information.

#### 3.2.3. Consumption of Foods Prepared at Home or Elsewhere

**Figure 3:**
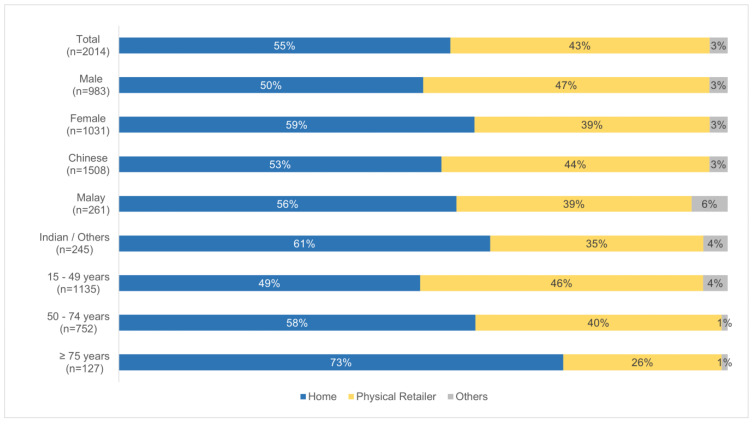
Breakdown of the consumption of food prepared at home or elsewhere, stratified by gender, race, and age group.

**Figure 4:**
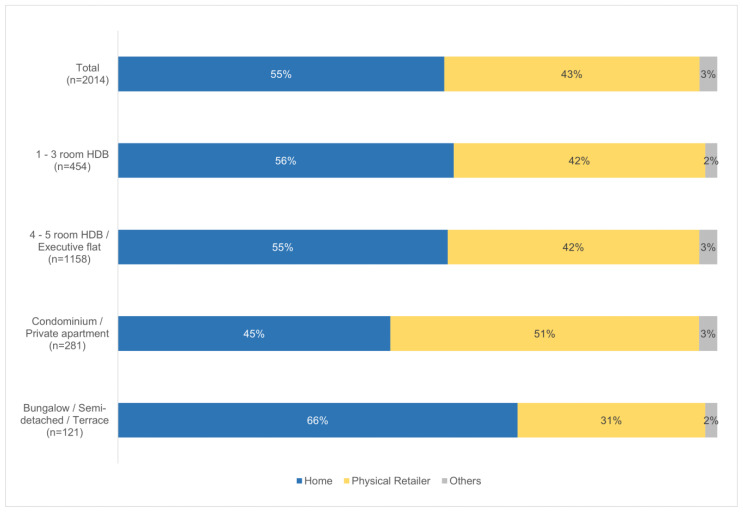
Breakdown of the consumption of food prepared at home or elsewhere, stratified by housing type.

Understanding food consumption patterns also includes knowing if the population tends to consume foods prepared at home or elsewhere. This was obtained through the food consumption survey, as respondents were also asked if the foods and beverages reported to be consumed were purchased or prepared at home. Figure 3 and Figure 4 show the breakdown of the consumption of foods prepared at home or elsewhere, and it is observed that among the different sub-groups analysed, individuals aged 75 years and above consumed the most home-prepared food (73%), followed by individuals living in bungalows/semi-detached/terraces (66%).

#### 3.2.4. Cooking Methods Adopted by the Singapore Population

A TDS includes foods that are representative of the population, in a consumption-ready state. Thus, knowledge of the cooking methods commonly adopted for different foods by the Singapore population was gathered in the study. Some examples of foods, along with their commonly adopted cooking methods, collected through the survey can be found in Table S5 of Supplementary Information.

**Figure 5:**
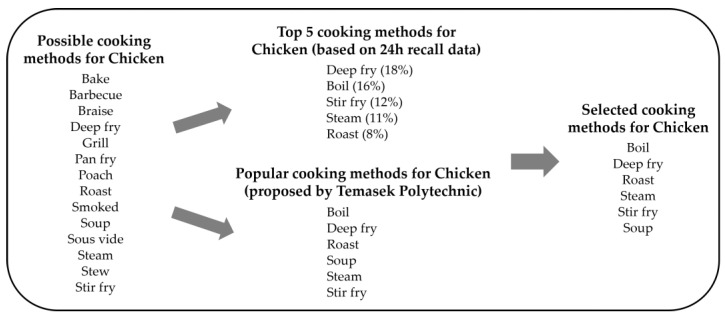
An example illustrating the selection process for the cooking methods of chicken.

Supplementing the findings from the survey, commonly adopted cooking methods for different foods were determined based on consultants’ advice, and Figure 5 illustrates an example of the process leading to the food-cooking method combinations for chicken. The list of food-cooking method combinations for the Singapore TDS can be found in Table S6 of Supplementary Information. 

### 3.3. Derivation of the TDS Food List and Sampling of Foods

#### 3.3.1. Derivation of the TDS Food List Based on Food Consumption Patterns of the Singapore Population and Chemical Hazards Included

The foods and beverages reported in the 24 h recall survey resulted in a sizeable list, and a set of criteria [1] was used for the selection of foods that are representative of the Singapore population’s diet. This set of criteria served as the basis of inclusion and allowed foods that contribute to 90% of the total food consumed (by weight), foods consumed by more than 10% of the consumers, as well as foods consumed by more than 10% of each race [36,37] to be included in Singapore’s TDS. Consequently, this led to the inclusion of foods consumed in the largest amounts and formed the bulk of the food list, as commonly consumed foods in the major food categories such as grains and grain-based products, meat and meat products, fish and seafood, vegetables and fruits were already mostly included. Tables S7 and S8 in Supplementary Information, respectively, show foods that contributed to 90% of the total food consumed and foods consumed by more than 10% of all consumers.

A multi-racial society is one of the distinctive characteristics of the Singapore population, comprising Chinese, Malay, Indian, and Others. Hence, based on the number of respondents surveyed from each race, foods consumed by more than 10% were included in the food list to ensure that foods primarily commonly consumed by a particular race were also captured (refer to Table S9 of Supplementary Information).

In addition to the inclusion of foods that are commonly consumed by the population, foods consumed in smaller amounts (by weight) but which may have a high contribution to dietary exposure (e.g., spices, dried foods) were also included (Table S10 of Supplementary Information). The final list of foods and beverages included in Singapore’s TDS can be found in Table S11 of Supplementary Information.

### 3.4. Chemical Concentrations Found in Foods

The mean concentrations for metals and processed contaminants studied in Singapore’s TDS by food category are shown in Table 4 and Table 5. Concentration data for the other chemical groups can be found in Tables S12 to S15 of Supplementary Information, and elsewhere [38,39,40].

## 4. Discussion

The number of chemicals that can potentially be found in foods is considerable. Therefore, in the current study, principles recommended by the WHO [2] were applied in the prioritization and selection of chemicals. For instance, one of the chemical groups included is contaminants formed during food processing. Singapore is known for its hawker culture, and the population is exposed to foods from different cultures and cuisines. Thus, the consumption of foods that are cooked using methods that involve high temperatures or longer cooking durations such as roasting, deep frying, frying, grilling, and baking are not uncommon, and the chemical contributions from processed contaminants should not be underestimated. Moreover, some of them such as acrylamide, furan, and nitrosamines are genotoxic and/or carcinogenic, underlining the need for monitoring and inclusion in the TDS. 

Chemicals subjected to an international convention were also considered for inclusion in Singapore’s TDS. For instance, the Stockholm convention is an international convention that publishes and maintains a list of Persistent Organic Pollutants (POPs) whose use should be eliminated, restricted, or reduced. Some examples of POPs in the list include pesticides such as hexachlorobenzene (HCB), lindane, aldrin, as well as environmental contaminants like polychlorinated biphenyls (PCBs), polychlorinated dibenzofurans (PCDFs), polychlorinated dibenzo-*p*-dioxins (PCDDs), perfluorooctane sulfonic acid (PFOS), and perfluorooctanoic acid (PFOA). These are persistent chemicals that can bioaccumulate and cause harmful effects to health; hence, the Singapore population’s baseline exposures to these chemicals would be monitored through the TDS.

The food consumption patterns of the Singapore population revealed that beverages, grain, and grain-based products consistently constituted about half of all sub-groups’ average consumption in a day (Figure 1 and Figure 2). These two food categories are more frequently consumed throughout the day, coupled with relatively larger amounts (based on weight) consumed as compared to other foods such as meats, fish, and vegetables. Among the races, a lower consumption of meat, fish, and seafood among Indians (refer to Figure 1) was not unexpected given that the Indian culture is often associated with its relatively high rate of vegetarianism or reduced consumption of meat in the diet [41,42]. Consequently, Indians’ consumption of milk and dairy products, as well as composite foods such as dhal, curry, and idli is significantly higher than the other races. However, in general, there were no significant differences in food consumption patterns across the races. The survey took place during the COVID-19 pandemic, over a 6-month period from August 2021 to January 2022, where the population’s food consumption patterns may have changed. 

Among the age groups studied, a higher consumption of grain and grain-based products, fish and seafood, as well as bakery products was observed among individuals aged 75 years and above, coupled with a lower consumption of meat (refer to Figure 2). This could likely be attributed to chewing and swallowing difficulties commonly observed among the elderly, accompanied by a loss of appetite [43], causing them to choose simpler foods for their meals such as porridge and bread. The higher consumption of fish similarly could be because it is soft and easy to chew [44], and serves as a source of lean protein. 

On the consumption of home-prepared meals, the higher occurrence among the elderly in Singapore (refer to Figure 3) was previously also observed in a cross-sectional study conducted in 2017 [45]. It is possible that the elderly may have mobility issues and tend to go out less, thereby leading to more frequent consumption of home-prepared meals. As for individuals staying in bungalows/semi-detached/terraces, the higher consumption of home-prepared meals as compared to those living in other housing types (refer to Figure 4) could be due to a higher socioeconomic status (SES) possessed by this group, thereby consisting of either a stay-home parent or a domestic worker that could prepare meals at home.

Based on the food consumption data collected from 24 h recall interviews, foods that met the set of criteria listed in Section 3.3.1 were sampled in Singapore’s TDS (refer to Table S11 of Supplementary Information). This approach enabled the inclusion of foods that are widely consumed, consumed in large amounts, or those that may potentially possess higher chemical levels, all of which affect dietary exposures. Singapore is a multi-racial society; hence, differences in the diet of consumers from the four major races are expected. The findings from the food consumption survey showed differences in foods typically consumed by consumers in each race (refer to Table S9 of Supplementary Information), but the overall consumption patterns remained generally similar (refer to Figure 1). For instance, among grains and grain-based products, the Chinese showed a higher consumption of rice noodle, while Indians showed a higher consumption of basmati rice, chapati, and thosai. Among vegetables, the Chinese showed a higher consumption of chye sim, bitter gourd, and xiao bai cai, while Indians showed a higher consumption of lentil, cauliflower, and brinjal. Among meat and meat products, mutton was found to be more commonly consumed by Malays and Indians, while pork was predominantly consumed by the Chinese and Others. These examples demonstrate the importance and relevance of capturing the commonly consumed foods of consumers from the four major races, so that the representative diet of the Singapore population can be accurately portrayed. Based on chemical hazards included in this study, the TDS food list also included some foods that may possess higher chemical concentrations even though they are not consumed in large amounts. Some examples include chilli powder, honey, seaweed, sea cucumber, oyster, and scallop [46,47,48,49,50]. Dried fruits were also included as the removal of moisture could lead to a higher concentration of chemicals.

Food preparation practices which include the removal of inedible parts, washing/cleaning of foods, and cutting prior to cooking were adopted in the process of preparing foods to a consumption-ready state. These are aspects that may differ across populations, and it is important that the preferences and habits of the Singapore population were mimicked to accurately reflect the population’s dietary exposures. In the preparation and cooking of foods in Singapore’s TDS, cooking oils and stainless-steel utensils, cookware, and equipment were used. The use of cooking oils in the food preparation step of a country’s TDS is an aspect that varies widely across different countries. For instance, China and France added oils, while New Zealand, Hong Kong, and Korea did not [25,26,27,51,52]. In Singapore’s TDS, findings from the food consumption survey revealed that the Singapore population tends to use cooking oil in the cooking of foods; hence, cooking oils were used to obtain a closer depiction of the way foods are prepared in Singapore. Furthermore, some cooking methods adopted in this study such as deep frying, pan frying, and stir frying require the use of cooking oils, without which the levels of processed contaminants may be underestimated, and the organoleptic properties of the cooked food would also be affected. 

While TDSs conducted in other countries often focus on the single most prevalent cooking method [53], this approach may not be representative of the Singapore diet. The hawker culture, accessibility of international cuisine, and multi-racial society are factors that influence the food consumption choices of the Singapore population. Many foods in Singapore are cooked in multiple ways, some of which lead to very different levels of contaminants, particularly processed contaminants. Hence, multiple cooking methods were adopted for applicable foods, to accurately reflect the Singapore population’s consumption habits and preferences. In addition, by incorporating a variety of cooking methods in the study, the effect of cooking method on the levels of processed contaminants could also be investigated subsequently. 

In developing the food list, other factors such as the possibility of the mapping of foods and food pooling were also considered. The food consumption survey gave rise to a long and diverse list of foods, and it was observed that some foods were occasionally consumed but similar in nature, e.g., tropical fruits, sauces, and some species of fish and seafood; hence, food mapping was carried out. As it is not possible to sample all foods, foods that can be mapped to other foods similar in terms of contamination or composition (refer to Table S10 of Supplementary Information) were also considered for inclusion in this study [24]. Food mapping has also been adopted by other countries such as Australia [24], Korea [53], and Hong Kong [26]. For instance, the Singapore TDS mapped radish and turnip to carrot, lemon to lime, and beef sausage to beef bacon. Mapped foods are less commonly consumed, but their contributions to the total dietary exposures can still be factored in by combining the amount consumed with the concentration found in the representative food. 

Pooling of foods is a key characteristic of TDS, and it was carried out at an individual food level in the current study. As opposed to pooling at a food group level, which would result in fewer samples to analyse, the flexibility to pair contamination levels with varying food consumption amounts observed across different population sub-groups can be retained through this approach, thereby improving the resolution and accuracy of the dietary exposure assessment for Singapore. 

In designing the sampling plan, the seasonality of foods and variation in import sources through the year was also considered. An analysis of Singapore’s variation in import quantity throughout the year 2020 revealed that for most foods, the supply from Singapore’s major importing countries was consistent throughout the year, except for some fruits and wild-caught fishes and seafoods. Hence, several types of fruits, fish, and seafood were sampled over two occasions, so as to maximise the inclusion of the variety available in the year. 

Metals are inorganic contaminants, and those included in Singapore’s TDS are naturally occurring and ubiquitous in the environment. Consequently, their presence was detected across many food categories, with aluminium, arsenic, chromium, lead, and nickel detected in all food categories tested. Fish and seafood, and fungi, seaweed frequently showed higher levels as compared to other food categories in this study (refer to Table 4). The levels of aluminium in this study were highest in RTE savouries, fish and seafood, and bakery products. Higher levels of aluminium found in RTE savouries were attributed to fish-based snacks such as fish skin, and ikan satay, while higher concentrations found in bakery products were likely due to the presence of alum-containing leavening agents. The latter finding is consistent with other countries such as New Zealand [25], UK [54], and Hong Kong [55]. Methylmercury has a tendency to bioaccumulate in aquatic organisms, and in the current study, only some species of fish and seafood possessed levels of total mercury higher than the detection limits of methylmercury and were consequently tested. TDSs conducted by other countries such as Australia [24], New Zealand [25], Hong Kong [55], and Germany [56] also mostly tested methylmercury in fish and seafood. Inorganic arsenic was detected in several food categories, with the highest levels found in fungi, seaweed, fish and seafood, and composite foods, followed by grains and grain-based products. Higher levels detected in these food categories were mostly expected, but the higher inorganic arsenic concentrations found in composite foods were likely because this food category comprised some grain-based products such as idli, pizza, and fried carrot cake. On the whole, the concentrations of metals found in Singapore’s TDS were comparable with other countries such as Hong Kong, China, Australia, USA, and France [24,55,57,58,59].

Among the mycotoxins and phytotoxins studied in this TDS, Aflatoxin M and tropane alkaloids were not detected, while pyrrolizidine alkaloids were only detected in honey. For Aflatoxin B and G, the highest concentrations were detected in legumes (1.16 ppb), followed by bakery products (0.95 ppb), RTE savouries (0.51 ppb), and sauces and condiments (0.48 ppb). Pyrrolizidine alkaloids were only detected in honey, which was not a surprising observation [50]. Veterinary drug residues were only found in meat and meat products as well as eggs and egg products in this study, and a detailed discussion can be found elsewhere [38]. The levels of pesticides in this study were generally low. 

On the presence of persistent organic pollutants, perfluorinated compounds were only detected in fish and seafood. A comparison of PFOS concentrations in selected foods revealed that the levels detected in our foods were comparable to or lower than other countries [60,61]. For instance, the PFOS concentration in tuna in Singapore’s TDS was 0.11 µg/kg, whereas that found in canned tuna in Australia’s 27th TDS was 0.07–0.095 µg/kg. The PFOS concentrations in Australia’s 27th TDS were 0.011–0.058 µg/kg in saltwater fish fillets, 0.96 µg/kg in fish in UK’s 2012 TDS, and 0.12 µg/kg in mackerel in Singapore’s TDS. As for dioxin and dioxin-like PCBs, they were detected in several food categories with the highest levels found in fish and seafood (Table S14 of Supplementary Information). This is aligned with the understanding that dioxins tend to accumulate in the fatty tissue of animals and are often found in meat, fish, and dairy products [62,63].

Levels of furan in this study were the highest in fats and oils (72.1 ppb), followed by RTE savouries (15.9 ppb), amongst which deep fried animal fat and popcorn showed the highest levels, followed by dark soy sauce. Previous studies conducted have shown higher furan levels in popcorn and soy sauce [32,64,65]. As for deep fried animal fat, the cooking duration and high temperature involved in the cooking process could have led to an increase in furan levels [66,67,68]. Heterocyclic aromatic amines (HAAs) were found to be the highest in RTE savouries (108 ppb), followed by fats and oils (82 ppb) in the current study. Among the HAAs tested, harman and norharman were most commonly detected, and this finding was also observed in Korea’s TDS [69]. Acrylamide levels in this study were highest in legumes (218.4 ppb), root and tubers (164.2 ppb), followed by RTE savouries (147.6 ppb). In particular, higher concentrations were found in roasted potato (478 ppb), black pepper (404 ppb), roasted sweet potato (377 ppb), stir fried pea (363 ppb), fried chips and cracker (349 ppb), and stir fried beansprout (324 ppb). Higher levels of acrylamide in vegetables are not uncommon, especially when deep fried, stir fried, or cooked in high temperatures, due to the presence of asparagines and reducing sugars [70,71,72]. The levels of 3-MCPD esters and glycidyl esters in this study were the highest in fats and oils (731 ppb) and RTE savouries (538 ppb). A detailed discussion on acrylamide, 3-MCPD esters and glycidyl esters tested in foods from this study has previously been published [39,40]. 

Bisphenols and plasticisers were generally not widely detected in this study. For bisphenols, higher levels were observed in sauces and condiments (0.075 ppm), fish and seafood (0.026 ppm), and RTE savouries (0.024 ppm). Amongst them, foods with higher concentrations were chilli powder, mussels, and canned sardines. Among the plasticisers analysed in this study, DEHP was the most commonly detected, with the highest levels found in kaya (0.22 ppm) and skimmed milk (0.17 ppm). 

There were some limitations in this study. The food consumption data were taken from 24 h recall interviews conducted in Singapore prior to the study. While a repeated 24 h recall method is widely used to obtain food consumption data [73], a food frequency questionnaire (FFQ) could also be conducted on the same group of respondents to complement the data obtained through the 24 h recall method. This two-pronged approach enables a more comprehensive set of food consumption data to be collected for the Singapore population and will be considered in future TDSs. 

In the screening of chemical hazards in Singapore’s TDS, food additives were not included. Other practical aspects such as testing capabilities were also taken into consideration when determining the chemicals to be included in this study. The limits of detection and quantification (LODs and LOQs) for some test methods could also be improved in future TDSs, to reduce the number of non-detects which could subsequently enhance the estimates of the population’s dietary exposures.

When planning the TDS, the seasonality of foods was considered using seasonal variation in source rather than supply. This led to difficulties in amassing the required number of samples for some foods, as they were not widely available across the island. While this did not invalidate the TDS results, it increased the time taken for sampling as more places had to be visited in order to purchase all the samples required. Future TDSs should consider seasonal variation in supply for all foods when determining the sampling plan. Alternatively, the sampling duration for the study could be extended to a 12-month period since the peak supply may only occur during specific months within a year. 

Changes in consumer behaviour largely driven by the COVID-19 pandemic have led to an increase in the online purchasing of groceries. To mimic consumer behaviour, online purchasing of samples was also conducted in this study. However, some foods such as fruits posed additional complications as their ripeness was an aspect that cannot be controlled when purchased through e-commerce platforms. Depending on the situation, either sample preparation was carried out a few days later or in extreme cases, some replacement samples were purchased instead. Although this did not affect the results, the varying degrees of ripeness across all samples at the point of sample preparation is something that could be improved in future editions of this study. For instance, fruits should be purchased from physical stores as much as possible in subsequent TDSs, so that the appropriate ripeness can be chosen, and all samples would be ripe at the point of sample preparation. 

## 5. Conclusions

A TDS provides an estimate of the population’s baseline dietary exposure to chemicals in commonly consumed foods and has been endorsed by the WHO as the most cost-effective method for evaluating a population’s exposure to chemicals. The TDS is also a standardised risk assessment tool that enables comparisons among countries. This paper showcased the study design and methodology undertaken for Singapore’s TDS. The main methodological choices on the chemicals and foods included, as well as the 24 h recall survey which served as the source of food consumption data in this study, were presented. The distinctive characteristics of the Singapore population were pivotal to key decisions made in this study, such as the choice to adopt a variety of cooking methods, and the coverage of diets of individuals across the four main races of Singapore. Chemical concentrations in foods sampled in Singapore’s TDS were also determined and it was found that levels were generally comparable with other countries’ levels and food categories with higher levels were identified. Subsequently, population dietary exposures to the range of chemicals will be assessed and chemicals that pose a health concern to the population will be identified, so that measures can be taken to lower the risk. 

With the Singapore TDS carried out from 2021 to 2023, subsequent cycles of this study will enable the monitoring of chemical levels from across the diet in the future. Measures taken to manage the population’s dietary exposures can also be evaluated and assessed for effectiveness. Future publications will cover the Singapore population’s dietary exposures to chemicals.

## Figures and Tables

**Table 1:** Chemical hazards included in Singapore’s TDS.

Chemical Group	Chemical Compounds
Heavy metals	Aluminium, Antimony, Arsenic, Cadmium, Chromium, Inorganic arsenic, Lead, Mercury, Methylmercury, Nickel, Tin
Persistent organic pollutants (POPs)	Perfluorinated compounds (PFAS)—PFHxA, PFHxS, PFNA, PFOA, PFOS
	Dioxins and dioxin-like polychlorinated biphenyls (PCBs)-Mono-ortho polychlorinated biphenyls, Non-ortho polychlorinated biphenyls, Polychlorinated dibenzodioxins (PCDDs), Polychlorinated dibenzofurans (PCDFs)
Pesticide residue	Carbamates, Dithiocarbamates, Herbicides, Neonicotinoids, Organochlorines, Organophosphorus, Pyrethroids, Strobilurins, Triazoles and imidazoles, Other pesticides
Veterinary drug residues	β-agonists, Coccidiostats, Fluoroquinolones, Macrolides
Mycotoxins	Aflatoxins
	Deoxynivalenol and its derivatives
	Ergot alkaloids
	Fumonisins
	Ochratoxins
	Zearalenones and its derivatives
Phytotoxins	Pyrrolizidine alkaloids, Tropane alkaloids
Acrylamide	Acrylamide
3-MCPD	3-MCPD
Esters of 3-MCPD and glycidol	3-MCPD esters, Glycidyl fatty acid esters
Furans	2-methylfuran, Furan
Heterocyclic aromatic amines (HAAs)	4,8-DiMeIQx, AαC, Harman, IQ, MeIQ, MeIQx, Norharman, PhIP
Nitrosamines	NDBA, NDEA, NDMA, NMOR, NPIP, NPYR
Polyaromatic hydrocarbons (PAHs)	Benz[a]anthracene, Benzo[a]pyrene, Benzo[b]fluoranthene, Chrysene
Plasticisers	Adipates, Phthalates
Bisphenols	BADGE, BPA, BPS
Selenium	Selenium

**Table 2:** Demographic breakdown of survey respondents by gender, age, housing, and health condition, compared against the 2020 resident population breakdown in Singapore.

	Survey Respondents (%)	Resident Population, Singapore, 2020 [13] (%)
Gender		
Female	1031 (51.2)	1,778,532 (51.5)
Male	983 (48.8)	1,677,472 (48.5)
Age (years)		
15–49	1135 (56.4)	1,955,112 (56.6)
50–74	752 (37.3)	1,285,928 (37.2)
≥75	127 (6.3)	214,964 (6.2)
Housing type		
1–3 room HDB ^1^	454 (22.5)	669,011 (19.4)
4–5 room HDB ^1^/Executive flat	1158 (57.5)	2,043,081 (59.1)
Condominium/Private apartment	281 (14.0)	493,477 (14.3)
Bungalow/Semi-detached/Terrace	121 (6.0)	217,535 (6.3)
Other types of dwelling	-	32,900 (1.0)

^1^ HDB: Housing Development Board (refers to public housing).

**Table 3:** Demographic breakdown of survey respondents by age and race, compared against the 2020 resident population breakdown in Singapore.

Survey Respondents (%)					Number of Individuals, Actual, 2020 [13] (%)			
	15–49 Years Old	50–74 Years Old	>75 Years Old	Total	15–49 Years Old	50–74 Years Old	>75 Years Old	Total
Chinese	832 (41.3)	575 (28.6)	101 (5.0)	1508 (74.9)	1,419,133 (41.1)	1,004,571 (29.1)	184,532 (5.3)	2,608,236 (75.5)
Malay	153 (7.6)	89 (4.4)	19 (0.9)	261 (13.0)	273,124 (7.9)	152,704 (4.4)	16,460 (0.5)	442,288 (12.8)
Indian	109 (5.4)	67 (3.3)	6 (0.3)	182 (9.0)	190,189 (5.5)	97,359 (2.8)	11,389 (0.3)	298,937 (8.6)
Others	41 (2.0)	21 (1.0)	1 (0.0)	63 (3.1)	72,666 (2.1)	31,294 (0.9)	2583 (0.1)	106,543 (3.1)
Total	1135 (56.4)	752 (37.3)	127 (6.3)	2014 (100)	1,955,112 (56.6)	1,285,928 (37.2)	214,964 (6.2)	3,456,004 (100)

**Table 4:** Mean concentrations of various metals, in ppm, for each food category in Singapore’s TDS.

	Mean Concentration (ppm)											
Food Category	Aluminium	Antimony	Arsenic	Inorganic Arsenic	Cadmium	Chromium	Lead	Methylmercury	Mercury	Nickel	Selenium	Tin
Beverages	3.860	0.001	0.003	-	0.020	0.056	0.010	-	-	0.139	0.006	11.000
Bakery products	29.319	0.011	0.012	-	0.010	0.080	0.007	-	-	0.195	0.106	0.195
Fish and seafood	41.982	0.006	2.566	0.042	0.190	0.095	0.078	0.214	0.101	0.116	0.560	0.036
Grains and grain-based products	2.978	-	0.032	0.025	0.010	0.038	0.006	-	-	0.116	0.046	-
Meat and meat products	1.232	0.007	0.030	0.011	0.021	0.063	0.007	-	-	0.047	0.160	0.084
Eggs and egg products	1.067	-	0.045	0.021	-	0.020	0.005	-	-	0.035	0.238	0.024
Fats and oils	0.655	-	-	-	-	0.020	0.005	-	-	0.027	0.242	0.016
Milk and dairy products	0.639	-	0.005	-	-	0.021	0.008	-	-	0.036	0.043	0.456
Fruit and fruit products	3.418	0.012	0.017	0.013	0.010	0.041	0.051	-	-	0.141	0.026	0.041
Tap water, drinking water	0.034	-	0.001	-	-	0.002	0.000	-	-	0.003	0.000	-
Confectionary	4.476	-	0.005	-	0.023	0.089	0.006	-	-	0.208	0.053	0.755
RTE savouries	42.903	-	0.157	-	0.016	0.109	0.057	-	0.026	1.083	0.145	0.080
Fungi, seaweed	29.979	0.027	2.535	0.063	0.154	0.149	0.050	-	-	0.118	0.034	10.941
Nuts and seeds (including spices)	9.943	-	0.018	-	0.077	0.058	0.015	-	-	2.470	0.174	-
Vegetable protein	5.958	-	0.035	-	0.027	0.090	0.011	-	-	0.207	0.197	0.021
Brassica vegetables	3.594	-	0.010	-	0.010	0.030	0.050	-	-	0.050	-	0.026
Fruiting vegetables	0.429	-	0.009	-	0.010	0.018	0.008	-	-	0.033	-	0.015
Leafy vegetables, herbs	18.200	0.008	0.017	0.015	0.019	0.028	0.020	-	-	0.041	0.020	0.023
Legumes	1.720	-	0.007	-	0.019	0.032	0.011	-	-	0.488	0.036	0.019
Root and tubers	1.011	-	0.012	-	0.017	0.016	0.011	-	-	0.118	0.021	1.262
Stalk, stem and bulb vegetables	4.197	-	0.009	-	0.023	0.034	0.055	-	-	0.088	0.016	12.563
Sauces and condiments	18.144	0.009	0.110	0.011	0.025	0.221	0.027	-	-	0.418	0.057	0.042
Composite foods	4.371	-	0.026	0.038	0.009	0.069	0.007	-	-	0.182	0.061	0.849

**Table 5:** Mean concentrations of processed contaminants, in ppb, for each food category in Singapore’s TDS.

	Mean Concentration (ppb)				
Food Category	Chloropropanols	Heterocyclic Aromatic Amines	Nitrosamines	Polycyclic Aromatic Hydrocarbons (sum)	Furan Compounds
Beverages	-	31.0	-	-	4.2
Bakery products	20.4	34.0	1.3	-	4.7
Fish and seafood	21.0	44.3	2.3	2.2	3.4
Grains and grain-based products	33.8	6.3	1.8	-	3.9
Meat and meat products	24.3	1.9	1.8	-	2.1
Eggs and egg products	22.3	0.6	3.2	-	-
Fats and oils	10.4	82.0	1.7	0.7	72.1
Milk and dairy products	5.1	0.8	1.2	-	4.4
Fruit and fruit products	3.6	5.9	1.4	0.6	1.8
Tap water, drinking water	-	-	-	-	-
Confectionary	-	7.3	-	-	5.0
RTE savouries	4.0	107.8	2.9	0.7	15.9
Fungi, seaweed	5.5	2.3	1.4	0.8	3.4
Nuts and seeds (including spices)	-	6.5	1.2	-	2.1
Vegetable protein	29.7	21.5	3.4	-	5.5
Brassica vegetables	10.8	14.6	1.1	0.6	2.9
Fruiting vegetables	6.0	2.2	1.4	0.6	7.7
Leafy vegetables, herbs	14.2	4.7	1.4	1.0	3.0
Legumes	4.9	23.8	1.3	-	3.8
Root and tubers	4.7	20.3	1.4	-	1.9
Stalk, stem and bulb vegetables	16.2	3.7	1.6	7.3	2.0
Sauces and condiments	13.4	80.1	1.8	3.6	7.1
Composite foods	12.8	8.6	1.2	-	2.6

## Data Availability

Data is contained within the article or supplementary material.

## Supplementary Materials

The following supporting information can be downloaded at: https://www.mdpi.com/article/10.3390/foods13040511/s1, Table S1: Glossary of terms used for food preparation and cooking; Table S2: Limits of detection (LODs) and quantification (LOQs) for test methods used in Singapore’s TDS; Table S3: Method validation parameters for test methods used in this study; Table S4: Foods and beverages with the accompanying consumption amounts of the population; Table S5: Commonly adopted cooking methods for selected foods, obtained through the survey; Table S6: Food-cooking method combinations for foods that require cooking; Table S7: Foods contributing to 90% of the total amount of food and beverages consumed by weight; Table S8: Foods consumed by more than 10% of all consumers; Table S9: Foods consumed by more than 10% of the four major races of the Singapore population; Table S10: Foods consumed in small amounts but may have high contribution to dietary exposure; Table S11: Foods and beverages included in Singapore’s TDS food list; Table S12: Mean concentrations of mycotoxins and phytotoxins, in ppb, for each food category in Singapore’s TDS; Table S13: Mean concentrations of pesticide residues, in ppb, for each food category in Singapore’s TDS; Table S14: Mean concentrations of persistent organic pollutants, for each food category in Singapore’s TDS; Table S15: Mean concentrations of food contact chemicals, in ppm, for each food category in Singapore’s TDS.
