# Supplementary material for: Singapore’s Total Diet Study (2021–2023): Study Design, Methodology, and Relevance to Ensuring Food Safety
Source: Foods. 2024 Feb 6;13(4):511. doi: 10.3390/foods13040511 (PMC10887509; doi:10.3390/foods13040511)
Supplement: Supplementary file 1 [file foods-13-00511-s001.zip › foods-2820168-supplementary/TDS Manuscript 1 (study design) [Suppl Info].pdf]

Supporting Information

Table S1. Glossary of terms used for food preparation and cooking

Term	Definition
Homogenise	Using a blender, blend and mix until a homogeneous mixture is obtained.
Mix	For liquid foods. Thoroughly stir the mixture of liquids into a consistent, indistinguishable layer.
Blanch	Cook in boiling water for 2 minutes (unless otherwise stated). Transfer to ice water immediately, drain immediately once cooled to 25°C.
Boil	Cook using boiling water.
Rinse	Lightly wash with water at room temperature to remove residues of dirt or impurities.
Wash	Thoroughly wash foods with water at room temperature to remove dirt and impurities.
Drain	Remove excess liquid using a colander.
Pan fry	Fry foods in frying pan on an induction cooker/ gas burner, using low to medium heat. Prior to frying, coat bottom of pan with a thin layer of oil (approximately 1 tablespoon) and heat up.
Stir fry	Fry foods in frying pan on an induction cooker/ gas burner, using medium heat (150°C) (unless otherwise specified) with frequent stirring and turning over. Prior to frying, coat bottom of pan with a thin layer of oil (approximately 1 tablespoon) and heat up.
Bake	Cook food in oven using convectional dry heat from 150 – 200°C
Brew	Mix coffee powder with hot water at 95 – 100°C
Braise	Cook large pieces of food submerged in a liquid till tender
Deep fry	Cook food submerged in large amounts of oil at 150 – 180°C
Grill	Cook food over a griller with intense heat
Half-boil	Cook eggs submerged in hot water at 95 – 100°C
Reconstitute	Add water to rehydrate to original state
Roast	Cook food in oven using convectional dry heat from 180 – 200°C
Soup	Boil a food with water for a prolonged period of time
Steam	Cook food using steam as the mode of heat transfer at 100°C
Stew	Cook small pieces of food submerged in a liquid till tender

Table S2. Limits of detection (LODs) and quantification (LOQs) for test methods used in Singapore's TDS

Chemical class	Chemical compound	Limit of Detection (ppb)	Limit of Quantitation / Reporting Limit (ppb)	Method of determination
Heavy metals	Arsenic	5	15	Standard deviation of the response and slope (LOD: 3.3, LOQ: 10)
	Arsenic, inorganic	30 (dry samples)<br>10 (wet samples)	90 (dry samples)<br>30 (wet samples)	
	Aluminium	100	300	
	Antimony	5	15	
	Cadmium	5	15	
	Chromium	5	15	
	Lead	5	15	
	Mercury	10	30	
	Methylmercury	50	150	
	Nickel	5	15	
	Selenium	10	30	
	Tin	10	30	
Mycotoxins	Zearalenone & its derivatives	2	5	Signal-to-noise ratio (3:1 for LOD, 10:1 for LOQ)
	Fumonisin B1, B2	2	5	
	Aflatoxin B1, G1	0.025	0.075	
	Aflatoxin B2, G2	0.0625	0.01875	
	Aflatoxin M1, M2	0.03 (general)<br>0.01 (liq. milk, reconst. formula)	0.1 (general)<br>0.03 (liq. milk, reconst. formula)	
	Ergot alkaloids	0.3	1	
	DON & its derivatives (15-acetyl-deoxynivalenol, 3-acetyl-deoxynivalenol, Deoxynivalenol)	2.5	5	
	Ochratoxin A	0.05	0.1	
Phytotoxins (Pyrrolizidine alkaloids)	Echimidine	0.34	1	
	Echimidine-N-Oxide	0.34	1	
	Heliotrine	0.34	1	
	Heliotrine-N-oxide	0.34	1	
	Intermedine	0.34	1	
	Intermedine-N-oxide	0.34	1	
	Lasiocarpine	0.34	1	
	Lasiocarpine-N-oxide	0.34	1	
	Lycopsamine	0.34	1	

Chemical class	Chemical compound	Limit of Detection (ppb)	Limit of Quantitation / Reporting Limit (ppb)	Method of determination
	Lycopsamine-N-oxide	0.34	1	
	Monocrotaline	0.34	1	
	Monocrotaline-N-oxide	0.34	1	
	Retrorsine	0.34	1	
	Retrorsine-N-oxide	3.4	10	
	Riddelline	0.34	1	
	Riddelline-N-Oxide	3.4	10	
	Senecionine	0.34	1	
	Senecionine-N-oxide	0.34	1	
	Seneciphylline	0.34	1	
	Seneciphylline-N-oxide	0.34	1	
	Senkirkine	0.34	1	
	Trichodesmine	0.34	1	
Phytotoxins (Tropane alkaloids)	Atropine	0.3	0.9	
	Scopolamine	0.3	0.9	
Pesticide residues	Dithiocarbamates	0	20	Signal-to-noise ratio (3:1 for LOD, 10:1 for LOQ)
	ETU	10	20	
	PTU	10	10	
	Multi-residues	0	5 - 10	
Processed contaminants (Chloropropanols)	3-Monochloropane-1,2-diol (3-MCPD)	1	3	Standard deviation of the response and slope (LOD: 3.3, LOQ: 10)
Processed contaminants (Heterocyclic aromatic amines)	4,8-DiMeIQx	0.03	0.1	
	AαC	0.03	0.1	
	Harman	0.03	0.1	
	IQ	0.03	0.1	
	MeIQ	0.03	0.1	
	MeIQx	0.03	0.1	
	Norharman	0.03	0.1	
	PhIP	0.03	0.1	
Processed contaminants (Polycyclic aromatic hydrocarbons)	Benz(a)anthracene	0.15	0.5	Signal-to-noise ratio (3:1 for LOD, 10:1 for LOQ)
	Benzo(a)pyrene	0.15	0.5	
	Benzo(b)fluoranthene	0.15	0.5	
	Chrysene	0.15	0.5	
Processed contaminants (Furan compounds)	2-methylfuran	0.3	1	Standard deviation of the response and slope (LOD: 3.3, LOQ: 10)
	Furan	0.3	1	
	N-Nitrosodibutylamine (NDBA)	0.3	1	

Chemical class	Chemical compound	Limit of Detection (ppb)	Limit of Quantitation / Reporting Limit (ppb)	Method of determination
Processed contaminants (Nitrosamines)	N-Nitrosodiethylamine (NDEA)	0.3	1	Signal-to-noise ratio (3:1 for LOD, 10:1 for LOQ)
	N-Nitrosodimethylamine (NDMA)	0.3	1	
	N-Nitrosomorpholine (NMOR)	0.3	1	
	N-Nitrosopiperidine (NPIP)	0.3	1	
	N-Nitrosopyrrolidine (NPYR)	0.3	1	
Persistent organic pollutants (Dioxins (PCDDs/PCDFs/ PCBs))	1,2,3,4,6,7,8-Heptachlorodibenzo-p-dioxin (HpCDD)	0.0010	0.0030	Standard deviation of the response and slope (LOD: 3.3, LOQ: 10)
	1,2,3,4,6,7,8-Heptachlorodibenzofuran (HpCDF)	0.0010	0.0030	
	1,2,3,4,7,8,9-Heptachlorodibenzofuran (HpCDF)	0.0010	0.0031	
	1,2,3,4,7,8-Hexachlorodibenzo-p-dioxin (HxCDD)	0.0004	0.0013	
	1,2,3,4,7,8-Hexachlorodibenzofuran (HxCDF)	0.0003	0.0010	
	1,2,3,6,7,8-Hexachlorodibenzo-p-dioxin (HxCDD)	0.0004	0.0011	
	1,2,3,6,7,8-Hexachlorodibenzofuran (HxCDF)	0.0004	0.0011	
	1,2,3,7,8,9-Hexachlorodibenzo-p-dioxin (HxCDD)	0.0004	0.0012	
	1,2,3,7,8,9-Hexachlorodibenzofuran (HxCDF)	0.0004	0.0012	
	1,2,3,7,8-Pentachlorodibenzo-p-dioxin (PeCDD)	0.0002	0.0006	
	1,2,3,7,8-Pentachlorodibenzofuran (PeCDF)	0.0003	0.0009	
	2,3,4,6,7,8-Hexachlorodibenzofuran (HxCDF)	0.0003	0.0010	
	2,3,4,7,8-Pentachlorodibenzofuran (PeCDF)	0.0002	0.0006	
	2,3,7,8-Tetrachlorodibenzo-p-dioxin (TCDD)	0.0002	0.0006	
	2,3,7,8-Tetrachlorodibenzofuran (TCDF)	0.0002	0.0005	

Chemical class	Chemical compound	Limit of Detection (ppb)	Limit of Quantitation / Reporting Limit (ppb)	Method of determination
	1,2,3,4,5,6,7,8-Octachlorodibenzo-p-dioxin (OCDD)	0.0119	0.0361	
	1,2,3,4,5,6,7,8-Octachlorodibenzofuran (OCDF)	0.0096	0.0290	
	PCB congener 101	0.0212	0.0636	
	PCB congener 105	0.0126	0.0381	
	PCB congener 114	0.0159	0.0482	
	PCB congener 118	0.0179	0.0541	
	PCB congener 123	0.0118	0.0359	
	PCB congener 126	0.0018	0.0055	
	PCB congener 138	0.0202	0.0605	
	PCB congener 153	0.0205	0.0614	
	PCB congener 156	0.0173	0.0524	
	PCB congener 157	0.0146	0.0443	
	PCB congener 167	0.0143	0.0434	
	PCB congener 169	0.0031	0.0095	
	PCB congener 180	0.0324	0.0971	
	PCB congener 189	0.0131	0.0398	
	PCB congener 28	0.0195	0.0584	
	PCB congener 52	0.0173	0.0519	
	PCB congener 77	0.0023	0.0070	
	PCB congener 81	0.0024	0.0072	
Persistent organic pollutants (Perfluorinated compounds)	Perfluorohexanesulfonic acid (PFHxS)	0.3	1	
	Perfluorononanoic acid (PFNA)	0.3	1	
	Perfluorooctanesulfonate acid (PFOS)	0.3	1	
	Perfluorooctanoic acid (PFOA)	0.3	1	
	PFAS (in water)	0.015	0.05	
Food contact chemicals (Bisphenols)	Bisphenol A	0.6	2	Signal-to-noise ratio (3:1 for LOD, 10:1 for LOQ)
	Bisphenol A diglycidyl ether	0.6	2	
	Bisphenol S	0.6	2	
Food contact chemicals (Plasticisers)	Butyl benzyl phthalate (BBP)	30	100	
	Di-2-ethylhexyl adipate (DEHA)	30	100	
	Di-2-ethylhexyl phthalate (DEHP)	30	100	
	Di-butyl phthalate (DBP)	30	100	
	Di-iso-decyl phthalate (DIDP)	100	300	
	Di-iso-nonyl phthalate (DINP)	100	300	
	Di-n-decyl phthalate (DNDP)	30	100	
	Di-n-octyl phthalate (DNOP)	30	100	

Table S3. Method validation parameters for test methods used in this study
<Refer to attachment>

Table S4. Foods and beverages with the accompanying consumption amounts of the population
<Refer to attachment>

Table S5. Commonly adopted cooking methods for selected foods, obtained through the survey

Food	Commonly adopted cooking methods[1]
Rice, white	Steam (62%), Boil (27%), Fry (5%)
Noodles, yellow	Boil (62%), Stir fry (30%)
Chicken, breast	Boil (26%), Stir fry (22%), Steam (18%), Roast (10%)
Chicken, wing	Deep fry (57%), Stir fry (12%)
Pork, belly	Stir fry (36%), Boil (21%), Braise (13%)
Duck	Braise (32%), Roast (27%), Boil (18%)
Cod	Steam (76%), Boil (12%)
Broccoli	Stir fry (56%), Boil (30%)
Lettuce	No preparation (80%), Boil (13%)
Spinach	Stir fry (51%), Boil (38%)

[1] Totals may not sum to 100% as cooking methods less than 5% are not shown here

Table S6. Food-cooking method combinations for foods that require cooking

Food category	Food	Cooking method
Brassica vegetables	Broccoli	Boil
Brassica vegetables	Broccoli	Stir fry
Brassica vegetables	Cabbage	Boil
Brassica vegetables	Cabbage	Stir fry
Brassica vegetables	Cauliflower	Boil
Brassica vegetables	Cauliflower	Stir fry
Brassica vegetables	Preserved / pickled vegetable	Boil
Composite foods	Carrot cake	Pan fry
Composite foods	Dhal	Boil
Composite foods	Orh luak	Pan fry
Composite foods	Putu mayam	Steam
Composite foods	You tiao	Deep fry
Eggs and egg products	Century egg	Boil
Eggs and egg products	Century egg	No preparation
Eggs and egg products	Egg	Boil
Eggs and egg products	Egg	Braise
Eggs and egg products	Egg	Half boil
Eggs and egg products	Egg	Pan fry
Eggs and egg products	Egg	Steam
Eggs and egg products	Egg tofu	Boil
Eggs and egg products	Egg tofu	Pan fry

Food category	Food	Cooking method
Eggs and egg products	Salted duck egg	Boil
Fats and oils	Animal fat	Deep fry
Fats and oils	Vegetable oil	Deep fry
Fats and oils	Vegetable oil	Stir fry
Fish and seafood	Anchovy	Deep fry
Fish and seafood	Anchovy	Pan fry
Fish and seafood	Anchovy	Soup
Fish and seafood	Canned sardine	Stew
Fish and seafood	Canned sardine	Stir fry
Fish and seafood	Catfish	Deep fry
Fish and seafood	Clam	Boil
Fish and seafood	Clam	Stir fry
Fish and seafood	Cockle	Boil
Fish and seafood	Cockle	No preparation
Fish and seafood	Cockle	Stir fry
Fish and seafood	Crab	Boil
Fish and seafood	Crab	Stir fry
Fish and seafood	Fish ball, fish cake and related product	Boil
Fish and seafood	Fish head	Soup
Fish and seafood	Fish nugget and related product	Bake
Fish and seafood	Fish nugget and related product	Deep fry
Fish and seafood	Fish roe	No preparation
Fish and seafood	Grouper	Steam
Fish and seafood	Kuning and related fishes	Deep fry
Fish and seafood	Lobster / crayfish	Boil
Fish and seafood	Lobster / crayfish	Stir fry
Fish and seafood	Mackerel and related fishes	Pan fry
Fish and seafood	Mussel	Steam
Fish and seafood	Mussel	Stir fry
Fish and seafood	Oyster	Boil
Fish and seafood	Oyster	No preparation
Fish and seafood	Oyster	Stir fry
Fish and seafood	Prawn / shrimp	Boil
Fish and seafood	Prawn / shrimp	Deep fry
Fish and seafood	Prawn / shrimp	Soup
Fish and seafood	Prawn / shrimp	Stir fry
Fish and seafood	Salmon	Pan fry
Fish and seafood	Salted fish and related product	Deep fry
Fish and seafood	Salted fish and related product	Stir fry
Fish and seafood	Scallop	Boil
Fish and seafood	Scallop	No preparation
Fish and seafood	Scallop	Soup
Fish and seafood	Scallop	Stir fry

Food category	Food	Cooking method
Fish and seafood	Sea cucumber	Boil
Fish and seafood	Seabass	Steam
Fish and seafood	Snapper	Steam
Fish and seafood	Squid / cuttlefish	Boil
Fish and seafood	Squid / cuttlefish	Stir fry
Fish and seafood	Squid ball and related product	Boil
Fish and seafood	Threadfin	Steam
Fish and seafood	Trout / Cod	Boil
Fish and seafood	Trout / Cod	Deep fry
Fish and seafood	Trout / Cod	Steam
Fish and seafood	Trout / Cod	Stew
Fish and seafood	Tuna	Braise
Fish and seafood	Tuna	Pan fry
Fruiting vegetables	Bitter gourd	Boil
Fruiting vegetables	Bitter gourd	Stir fry
Fruiting vegetables	Bottle gourd	Stir fry
Fruiting vegetables	Brinjal	Boil
Fruiting vegetables	Brinjal	Steam
Fruiting vegetables	Brinjal	Stir fry
Fruiting vegetables	Capsicum	Boil
Fruiting vegetables	Capsicum	Stir fry
Fruiting vegetables	Chilli	Boil
Fruiting vegetables	Chilli	No preparation
Fruiting vegetables	Chilli	Stir fry
Fruiting vegetables	Cucumber	No preparation
Fruiting vegetables	Ladies finger	Boil
Fruiting vegetables	Ladies finger	Stir fry
Fruiting vegetables	Tomato	Boil
Fruiting vegetables	Tomato	No preparation
Fruiting vegetables	Tomato	Stir fry
Fruiting vegetables	Winter melon	Boil
Fruiting vegetables	Zucchini	Stir fry
Fungi, seaweed	Black jelly fungus	Boil
Fungi, seaweed	Canned mushroom	Boil
Fungi, seaweed	Dried mushroom	Boil
Fungi, seaweed	Fresh mushroom	Boil
Fungi, seaweed	Fresh mushroom	Stir fry
Fungi, seaweed	Seaweed	Boil
Fungi, seaweed	Seaweed	Soup
Fungi, seaweed	White jelly fungus	Boil
Grains and grain-based products	Barley	Soup
Grains and grain-based products	Brown rice	Steam
Grains and grain-based products	Brown rice	Stir fry

Food category	Food	Cooking method
Grains and grain-based products	Buckwheat noodle	Boil
Grains and grain-based products	Coconut rice	Steam
Grains and grain-based products	Corn	Boil
Grains and grain-based products	Glutinous rice	Steam
Grains and grain-based products	Instant noodle	Boil
Grains and grain-based products	Noodles, wheat	Boil
Grains and grain-based products	Oat	Boil
Grains and grain-based products	Pasta	Boil
Grains and grain-based products	Rice noodle	Boil
Grains and grain-based products	Rice noodle	Stir fry
Grains and grain-based products	Udon	Boil
Grains and grain-based products	Vermicelli	Boil
Grains and grain-based products	White basmati rice	Boil
Grains and grain-based products	White basmati rice	Steam
Grains and grain-based products	White japonica rice	Steam
Grains and grain-based products	White jasmine rice	Steam
Grains and grain-based products	White jasmine rice	Stir fry
Grains and grain-based products	Yellow and egg noodle	Boil
Leafy vegetables, herbs	Bai cai	Boil
Leafy vegetables, herbs	Bai cai	Stir fry
Leafy vegetables, herbs	Basil	Stir fry
Leafy vegetables, herbs	Bayam	Boil
Leafy vegetables, herbs	Bayam	Stir fry
Leafy vegetables, herbs	Chinese celery	Boil
Leafy vegetables, herbs	Chinese celery	Stir fry
Leafy vegetables, herbs	Chye sim	Boil
Leafy vegetables, herbs	Chye sim	Stir fry
Leafy vegetables, herbs	Coriander	Boil
Leafy vegetables, herbs	Coriander	No preparation
Leafy vegetables, herbs	Coriander	Stir fry
Leafy vegetables, herbs	Kailan	Boil
Leafy vegetables, herbs	Kailan	Stir fry
Leafy vegetables, herbs	Kale	Boil
Leafy vegetables, herbs	Kale	Stir fry
Leafy vegetables, herbs	Kang kong	Boil
Leafy vegetables, herbs	Kang kong	Stir fry
Leafy vegetables, herbs	Lettuce	Boil
Leafy vegetables, herbs	Lettuce	No preparation
Leafy vegetables, herbs	Lettuce	Stir fry
Leafy vegetables, herbs	Malabar	Boil
Leafy vegetables, herbs	Malabar	Stir fry
Leafy vegetables, herbs	Mint leaf	No preparation
Leafy vegetables, herbs	Nai bai	Boil

Food category	Food	Cooking method
Leafy vegetables, herbs	Nai bai	Stir fry
Leafy vegetables, herbs	Pandan leaf	Soup
Leafy vegetables, herbs	Spinach	Boil
Leafy vegetables, herbs	Spinach	Stir fry
Leafy vegetables, herbs	Sweet potato leaf	Boil
Leafy vegetables, herbs	Sweet potato leaf	Stir fry
Leafy vegetables, herbs	Watercress	Boil
Leafy vegetables, herbs	Xiao bai cai	Boil
Leafy vegetables, herbs	Xiao bai cai	Stir fry
Legumes	Beansprout	Boil
Legumes	Beansprout	Stir fry
Legumes	Dried bean	Boil
Legumes	Fermented soybean product	Stir fry
Legumes	Lentil	Boil
Legumes	Lentil	Stir fry
Legumes	Long bean	Stir fry
Legumes	Pea	Boil
Legumes	Pea	Stir fry
Legumes	Pea sprout	Boil
Legumes	Pea sprout	Stir fry
Legumes	Soy beancurd, tofu, tau kwa, taupok, tempeh	Boil
Legumes	Soy beancurd, tofu, tau kwa, taupok, tempeh	Pan fry
Legumes	Soybean	Soup
Meat and meat products	Beef bacon	Pan fry
Meat and meat products	Beef, no fat	Boil
Meat and meat products	Beef, no fat	Grill
Meat and meat products	Beef, no fat	Pan fry
Meat and meat products	Beef, no fat	Stew
Meat and meat products	Beef, no fat	Stir fry
Meat and meat products	Beef, with fat	Boil
Meat and meat products	Beef, with fat	Grill
Meat and meat products	Beef, with fat	Pan fry
Meat and meat products	Beef, with fat	Stew
Meat and meat products	Beef, with fat	Stir fry
Meat and meat products	Chicken	Boil
Meat and meat products	Chicken	Deep fry
Meat and meat products	Chicken	Roast
Meat and meat products	Chicken	Soup
Meat and meat products	Chicken	Steam
Meat and meat products	Chicken	Stir fry
Meat and meat products	Chicken ham, bologna	No preparation
Meat and meat products	Chicken nugget	Bake

Food category	Food	Cooking method
Meat and meat products	Chicken nugget	Deep fry
Meat and meat products	Chicken sausage, hot dog, cocktail	Boil
Meat and meat products	Chicken sausage, hot dog, cocktail	Pan fry
Meat and meat products	Chinese sausage	Stir fry
Meat and meat products	Duck	Boil
Meat and meat products	Duck	Braise
Meat and meat products	Duck	Roast
Meat and meat products	Mutton, no fat	Boil
Meat and meat products	Mutton, no fat	Stew
Meat and meat products	Mutton, no fat	Stir fry
Meat and meat products	Pork bacon	Pan fry
Meat and meat products	Pork ham, bologna	No preparation
Meat and meat products	Pork luncheon meat	Deep fry
Meat and meat products	Pork luncheon meat	No preparation
Meat and meat products	Pork luncheon meat	Pan fry
Meat and meat products	Pork meat ball	Boil
Meat and meat products	Pork organ	Boil
Meat and meat products	Pork, no fat	Boil
Meat and meat products	Pork, no fat	Roast
Meat and meat products	Pork, no fat	Stir fry
Meat and meat products	Pork, with fat	Boil
Meat and meat products	Pork, with fat	Roast
Meat and meat products	Pork, with fat	Stir fry
Meat and meat products	Prawn meat ball	Boil
Nuts and seeds (including spices)	Peanut	Deep fry
Root and tubers	Carrot	Boil
Root and tubers	Carrot	No preparation
Root and tubers	Carrot	Stir fry
Root and tubers	French fries	Deep fry
Root and tubers	Lotus root	Soup
Root and tubers	Lotus root	Stir fry
Root and tubers	Potato	Bake
Root and tubers	Potato	Boil
Root and tubers	Potato	Deep fry
Root and tubers	Potato	Roast
Root and tubers	Potato	Stir fry
Root and tubers	Pumpkin	Boil
Root and tubers	Sweet potato	Bake
Root and tubers	Sweet potato	Boil
Root and tubers	Sweet potato	Roast
Root and tubers	Sweet potato	Steam
Root and tubers	Yam	Boil
Root and tubers	Yam	Steam

Food category	Food	Cooking method
Sauces and condiments	Sambal chilli / belacan	Stir fry
Sauces and condiments	Soy sauce, light	Boil
Sauces and condiments	Soy sauce, light	No preparation
Stalk, stem and bulb vegetables	Asparagus	Boil
Stalk, stem and bulb vegetables	Bamboo shoot	Boil
Stalk, stem and bulb vegetables	Celery	Boil
Stalk, stem and bulb vegetables	Celery	Stir fry
Stalk, stem and bulb vegetables	Dried lily bulb	Boil
Stalk, stem and bulb vegetables	Garlic	Soup
Stalk, stem and bulb vegetables	Garlic	Stew
Stalk, stem and bulb vegetables	Garlic	Stir fry
Stalk, stem and bulb vegetables	Ginger	Soup
Stalk, stem and bulb vegetables	Ginger	Steam
Stalk, stem and bulb vegetables	Ginger	Stir fry
Stalk, stem and bulb vegetables	Lily bulb	Stir fry
Stalk, stem and bulb vegetables	Lily bulb	Stir fry
Stalk, stem and bulb vegetables	Onion	Boil
Stalk, stem and bulb vegetables	Onion	Stir fry
Stalk, stem and bulb vegetables	Spring onion	No preparation
Stalk, stem and bulb vegetables	Turmeric	Boil
Vegetable protein	Mock meat (gluten based)	Braise
Vegetable protein	Mock meat (gluten based)	Stir fry
Vegetable protein	Mock meat (soy based)	Braise
Vegetable protein	Mock meat (soy based)	Deep fry
Vegetable protein	Mock meat (soy based)	Stir fry

Table S7. Foods contributing to 90% of the total amount of food and beverages consumed by weight

Food category	Food
Beverages	Alcohol, beer
	Alcohol, red wine
	Carbonated soft drink
	Instant coffee
	Non-carbonated sweetened beverage
	Isotonic sport drinks
	Juice drink (manufactured, excluding freshly prepared juices)
	Sugarcane juice
	Malted, cereal or chocolate drink
	Cocoa powder
	Freshly brewed coffee

Food category	Food
Milk and dairy products	Butter, regular
	Cheese, low fat
	Cheese, regular
	Fermented milk
	Ice cream
	Milk, condensed
	Milk, powdered, low fat
	Milk, powdered, regular
	Milk, powdered, skimmed
	Milk, low fat
	Milk, regular

Food category	Food
	Green tea and chinese tea
	Red (english) tea
	Bottled water
	Milk tea
Bakery products	Biscuit
	Cake
	Kueh, no coconut
	Kueh, with coconut
	Pancake
	Pastry
	Puff and pie
	Savoury bun (bao)
	Sweet bun (bao)
	White bread
	Wholemeal bread
Fish and seafood	Crab
	Threadfin (ngor he)
	Snapper
	Seabass
	Grouper
	Catfish
	Squid / cuttlefish
	Salmon
	Prawn / shrimp
	Canned tuna
	Fish ball, fish cake and related product
	Mackerel and related fishes (e.g. batang)
	Fish head
Grains and grain-based products	Barley
	Breakfast cereal
	Coconut rice
	Corn
	Glutinous rice
	Rice noodle
	Noodles, wheat (e.g. ban mian)
	Instant noodle
	Yellow and egg noodle
	Vermicelli
	Udon
	Oat
	Pasta

Food category	Food
	Milk, skimmed
	Yoghurt, low fat
	Yoghurt, regular
	Creamer
Brassica vegetables	Broccoli
	Cabbage
	Cauliflower
Fruiting vegetables	Bitter gourd
	Bottle gourd
	Brinjal
	Capsicum
	Cucumber
	Ladies finger
	Tomato
	Winter melon
	Zucchini
Leafy vegetables, herbs	Bai cai
	Bayam
	Chinese celery
	Chye sim
	Kailan
	Kale
	Kang kong
	Lettuce
	Malabar
	Nai bai
	Spinach
	Sweet potato leaf
	Watercress
	Xiao bai cai
Legumes	Beancurd dessert
	Beansprout
	Lentil
	Long bean
	Pea
	Soybean
	Soy beancurd, tofu, tau kwa, taupok, tempeh
	Soy milk
Root and tubers	Carrot
	French fries
	Potato

Food category	Food
	Roti prata
	Thosai
	White jasmine rice
	White basmati rice
	White japonica rice
	Brown rice
Meat and meat products	Beef, no fat
	Beef, with fat
	Chicken
	Chicken ham, bologna
	Chicken nugget
	Chicken sausage, hot dog, cocktail
	Duck
	Pork ham, bologna
	Pork meat ball
	Pork, no fat
	Pork, with fat
	Pork luncheon meat
Eggs and egg products	Egg
	Egg tofu
Fungi, seaweed	Fresh mushroom
	Dried mushroom
	Canned mushroom
Nuts and seeds	Peanut butter
	Sambal chilli / belacan
Sauces and condiments	Soy sauce, light
	Sugar, white
	Tomato sauce
	Vegetable oil
Fats and oils	Olive oil
Tap water, drinking water	Water

Food category	Food
	Pumpkin
	Sweet potato
Stalk, stem and bulb vegetables	Celery
	Garlic
	Ginger
	Onion
	Spring onion
Fruit and fruit products	Apple
	Avocado
	Banana
	Blueberry
	Coconut milk
	Coconut water
	Grape
	Guava
	Kaya
	Kiwi
	Lime / calamansi
	Mango
	Melon
	Orange
	Papaya
	Pear
	Pineapple
	Watermelon
Composite foods	Dhal
	Idli
	Mushroom soup (canned, instant, prepared)
	You tiao
	Meat dumpling
	Curry gravy (meat)
	Curry gravy (fish)
	Fried carrot cake
	Boba (tapioca pearl)

Table S8. Foods consumed by more than 10% of all consumers

Food category	Food
Beverages	Alcohol, white wine
	Carbonated soft drink
	Freshly brewed coffee
	Instant coffee

Food category	Food
Vegetables	Basil
	Beansprout
	Broccoli
	Cabbage

Food category	Food
	Malted, cereal or chocolate drink
	Red (english) tea
Bakery products	Biscuit
	Sweet bun (bao)
	White bread
	Wholemeal bread
Fish and seafood	Fish ball, fish cake and related product
	Prawn / shrimp
	Snapper
Grains and grain-based products	Brown rice
	Instant noodle
	Rice noodle
	White jasmine rice
	Yellow and egg noodle
Meat and meat products	Beef
	Chicken
	Chinese sausage (eg. lup cheong)
	Pork
Eggs and egg products	Egg
Fats and oils	Vegetable oil
Milk and dairy products	Cheese, regular
	Butter, regular
	Milk, condensed
	Milk, regular
Fruit and fruit products	Apple
	Coconut milk
	Lime / calamansi
	Orange
Tap water, drinking water	Tap water
Fungi, seaweed	Dried mushroom
	Fresh mushroom
RTE savouries	Roasted nut snack

Food category	Food
	Capsicum
	Carrot
	Chilli
	Chye sim
	Coriander
	Cucumber
	Fermented soybean product
	Garlic
	Ginger
	Lettuce
	Onion
	Pandan leaf
	Pea
	Potato
	Soy beancurd, tofu, tau kwa, taupok, tempeh
	Spring onion
	Tomato
	Xiao bai cai
Sauces and condiments	Chilli powder
	Chilli sauce
	Fish sauce
	Oil, sesame
	Oyster sauce
	Pepper, black
	Pepper, white, powder
	Salt
	Sambal chilli / belacan
	Soy sauce, dark
	Soy sauce, light
	Sugar, white
	Tomato sauce
Composite foods	Curry gravy (meat)
	Meat dumpling

Table S9. Foods consumed by more than 10% of the four major races of the Singapore population

Food category	Food	Consumed by more than 10%			
		Chinese	Malay	Indian	Others
Beverages	Carbonated soft drink	√	√	√	√
	Freshly brewed coffee	√	√	√	√
	Instant coffee	√	√	√	√
	Malted, cereal or chocolate drink	√	√	√	
	Milk tea		√		√
	Red (english) tea	√	√	√	√
Bakery products	Biscuit		√	√	√
	White bread	√	√	√	√
	Wholemeal bread	√	√	√	√
Fish and seafood	Fish ball, fish cake and related product	√	√		√
	Prawn, pink	√			
	Salmon	√			
Grains and grain-based products	Breakfast cereal				√
	Brown rice	√		√	
	Chapati			√	
	Instant noodle			√	
	Oat	√		√	
	Pasta				√
	Rice noodle	√	√		√
	Roti prata		√	√	√
	Thosai			√	
	White basmati rice		√	√	√
	White japonica rice				√
	White jasmine rice	√	√	√	√
	Yellow and egg noodle	√	√	√	√
Meat and meat products	Beef		√		√
	Chicken	√	√	√	√
	Chinese sausage (eg. lup cheong)	√	√		√
	Mutton		√	√	
	Pork, no fat	√			√
	Pork, with fat	√			√
Eggs and egg products	Egg	√	√	√	√
Fats and oils	Olive oil				√
	Vegetable oil	√	√	√	√
Nuts and seeds (including spices)	Peanut		√		√
	Peanut butter		√		√
Milk and dairy products	Butter	√	√	√	√
	Cheese	√	√		√
	Milk, condensed	√	√	√	√

		Consumed by more than 10%			
**Food category**	**Food**	**Chinese**	**Malay**	**Indian**	**Others**
	Milk, powdered			√	
	Milk, regular		√	√	√
	Milk, skimmed				√
	Yogurt			√	
Fruit and fruit products	Apple	√		√	√
	Banana		√	√	
	Coconut flesh			√	
	Coconut milk		√	√	√
	Kaya	√			√
	Lime / calamansi	√	√	√	√
	Pineapple				√
Tap water, drinking water	Tap water	√	√	√	√
Vegetables	Beansprout	√	√	√	√
	Bitter gourd	√			
	Brinjal			√	√
	Broccoli	√		√	√
	Cabbage	√	√	√	√
	Carrot	√	√	√	√
	Cauliflower			√	
	Chilli	√	√	√	√
	Chye sim	√			√
	Coriander	√	√	√	√
	Cucumber	√	√	√	√
	Garlic	√	√	√	√
	Ginger	√	√	√	√
	Kang kong				√
	Ladies finger			√	
	Lentil			√	
	Lettuce	√	√		√
	Long bean			√	√
	Mint leaf				√
	Onion	√	√	√	√
	Pandan leaf	√	√	√	√
	Pea			√	
	Potato	√	√	√	√
	Soy beancurd, tofu, tau kwa, taupok, tempeh	√	√		√
	Spinach	√		√	
	Spring onion	√	√	√	√
	Tomato	√	√	√	√
	Turmeric		√	√	√
	Xiao bai cai	√			

Food category	Food	Consumed by more than 10%			
		Chinese	Malay	Indian	Others
Sauces and condiments	Chilli powder		√	√	√
	Chilli sauce	√	√	√	√
	Fish sauce	√	√	√	√
	Mayonnaise		√		√
	Oil, sesame	√	√	√	√
	Oyster sauce	√			√
	Pepper, black				√
	Pepper, white, powder	√	√		√
	Salt	√	√	√	√
	Sambal chilli/belacan		√	√	
	Soy sauce, dark	√	√	√	√
	Soy sauce, light	√	√	√	√
	Sugar, white	√	√	√	√
	Tomato sauce			√	√
	Vinegar				√
Composite foods	Curry gravy (meat)		√	√	√
	Curry gravy (fish)		√	√	
	Dhal			√	√
	Idli			√	

Table S10. Foods consumed in small amounts but may have high contribution to dietary exposure

Food category	Food
Brassica vegetables	Preserved / pickled vegetable
Stalk, stem and bulb vegetables	Dried lily bulb
	Lily bulb
	Asparagus
	Bamboo shoot
Legumes	Dried bean
	Pea sprout
Root and tubers	Yam
	Lotus root
Fungi, seaweed	Dried seaweed
	Seaweed
	Black jelly fungus
	White jelly fungus
Nuts and seeds	Sesame seed
Fruit and fruit products	Dried raisins
	Dried apricot
	Dried dates
	Dried mango
	Dried prunes

Food category	Food
	Aloe vera
	Dragonfruit
	Durian
	Jackfruit
	Longan
	Mandarin orange
	Mangosteen
	Peach
	Persimmon
	Plum
	Pomelo
	Rambutan
	Soursop
	Starfruit
	Strawberry
	Water chestnut
	Jam
Sauces and condiments	Chilli powder
	MSG
	Satay sauce
	Cream sauce
	Cheese sauce
	Mustard sauce
	Thousand island sauce
	Teriyaki sauce
	Wasabi
Fats and oils	Animal fat
Eggs and egg products	Salted duck egg
	Century egg
RTE savouries	Fish based snack
	Fried chips and cracker
	Popcorn
	Roasted nut snack
Meat and meat products	Pork organ
	Pork bacon
	Prawn meat ball
	Beef bacon
Milk and dairy products	Margarine
Bakery products	Bun, custard
Confectionary	Chocolate
	Honey
	Candy / sweet
	Dairy pudding

Food category	Food
	Jelly
	Syrup
Fish and seafood	Clam
	Cockle
	Lobster / crayfish
	Mussel
	Oyster
	Sea cucumber
	Scallop
	Anchovy
	Salted fish and related product
	Trout / Cod
	Tuna
	Kuning and related fishes
	Canned sardine
	Fish nugget and related product
	Fish roe
	Squid ball and related product
Grains and grain-based products	Rice dumpling
	Buckwheat noodle
Beverages	Energy drinks (e.g. red bull)
Vegetable protein	Mock meat (gluten, soy-based)
Composite foods	Pizza
	Fried oyster omelette (orh luak)
	Putu mayam
	Kimchi

Table S11. Foods and beverages included in Singapore's TDS food list

Food Category	Foods
Bakery products	Biscuit
	Bun, custard
	Cake
	Kueh, no coconut
	Kueh, with coconut
	Pancake
	Pastry
	Puff and pie
	Savoury bun (bao)
	Sweet bun (bao)
	White bread
	Wholemeal bread
	Alcohol, Beer

Food Category	Foods
Fruit and fruit products	Aloe vera
	Apple◊
	Avocado◊
	Banana
	Blueberry◊
	Coconut flesh
	Coconut milk
	Coconut water
	Dragonfruit
	Dried apricot
	Dried dates
	Dried mango
	Dried prunes

Food Category	Foods
Beverages (including bottled water)	Alcohol, Red Wine
	Alcohol, White Wine
	Bottled water
	Carbonated soft drink
	Cocoa powder
	Energy drinks
	Freshly brewed coffee
	Green tea and chinese tea
	Instant coffee
	Isotonic sport drinks
	Juice drink
	Malted, cereal or chocolate drink
	Milk tea
	Non-carbonated sweetened beverage
	Red (english) tea
	Sugarcane juice
Tap water, drinking water	Tap water
Composite foods	Boba (tapioca pearl)
	Carrot cake (菜头粿)
	Curry gravy (fish)
	Curry gravy (meat)
	Dhal
	Idli
	Kimchi
	Meat dumpling
	Mushroom soup
	Orh luak
	Pizza
	Putu mayam
	You tiao
Confectionary	Candy / sweet
	Chocolate
	Dairy pudding
	Honey
	Jelly
	Syrup
Eggs and egg products	Century egg
	Egg
	Egg tofu

Food Category	Foods
	Dried raisins
	Durian[◊]
	Grape[◊]
	Guava
	Jackfruit
	Jam
	Kaya
	Kiwi[◊]
	Lime / calamansi
	Longan
	Mandarin orange[◊]
	Mango[◊]
	Mangosteen[◊]
	Melon
	Orange[◊]
	Papaya
	Peach[◊]
	Pear[◊]
	Persimmon[◊]
	Pineapple
	Plum[◊]
	Pomelo[◊]
	Rambutan[◊]
	Soursop
	Starfruit[◊]
	Strawberry[◊]
	Water chestnut
	Watermelon
Fish and seafood	Anchovy[◊]
	Canned sardine
	Canned tuna
	Catfish
	Clam
	Cockle[◊]
	Crab
	Fish ball, fish cake and related product
	Fish head, boiled
	Fish nugget and related product
	Fish roe

Food Category	Foods
	Salted duck egg
Fats and oils	Animal fat
	Olive oil
	Vegetable oil
Grains and grain-based products	Barley[◊]
	Breakfast cereal
	Brown rice
	Buckwheat noodle
	Chapati
	Coconut rice
	Corn
	Glutinous rice
	Instant noodle
	Noodles, wheat
	Oat
	Pasta
	Rice dumpling[◊]
	Rice noodle
	Roti prata
	Thosai
	Udon
	Vermicelli[◊]
	White basmati rice
	White japonica rice
	White jasmine rice
	Yellow and egg noodle
Fungi, seaweed	Black jelly fungus
	Canned mushroom
	Dried mushroom
	Dried seaweed
	Fresh mushroom
	Seaweed[◊]
	White jelly fungus[◊]
Brassica vegetables	Broccoli[◊]
	Cabbage
	Cauliflower
	Preserved / pickled vegetable
Fruiting vegetables	Bitter gourd
	Bottle gourd

Food Category	Foods
	Grouper
	Kuning and related fishes
	Lobster / crayfish
	Mackerel and related fishes
	Mussel[◊]
	Oyster[◊]
	Prawn / shrimp
	Salmon
	Salted fish and related product[◊]
	Scallop[◊]
	Sea cucumber[◊]
	Seabass
	Snapper
	Squid / cuttlefish
	Squid ball and related product
	Threadfin
	Trout / Cod[◊]
	Tuna[◊]
Meat and meat products	Beef bacon
	Beef, no fat
	Beef, with fat
	Chicken
	Chicken ham, bologna
	Chicken nugget
	Chicken sausage, hot dog, cocktail
	Chinese sausage
	Duck
	Mutton, no fat
	Pork bacon
	Pork ham, bologna
	Pork luncheon meat
	Pork meat ball
	Pork organ
	Pork, no fat
	Pork, with fat
	Prawn meat ball
Milk and dairy products	Butter, regular
	Cheese, low fat
	Cheese, regular

Food Category	Foods
	Brinjal
	Capsicum
	Chilli
	Cucumber
	Ladies finger[◊]
	Tomato
	Winter melon
	Zucchini
Leafy vegetables, herbs	Bai cai
	Basil
	Bayam
	Chinese celery
	Chye sim
	Coriander
	Kailan
	Kale
	Kang kong
	Lettuce
	Malabar
	Mint leaf
	Nai bai
	Pandan leaf
	Spinach
	Sweet potato leaf
	Watercress
	Xiao bai cai
Legumes	Beancurd Dessert
	Beansprout
	Dried bean
	Fermented soybean product
	Lentil
	Long bean
	Pea
	Pea sprout
	Soy beancurd, tofu, tau kwa, taupok, tempeh
	Soy milk
	Soybean
Root and tubers	Carrot
	French fries

Food Category	Foods
	Creamer
	Fermented milk
	Ice cream
	Margarine
	Milk, condensed
	Milk, low fat
	Milk, powdered, low fat
	Milk, powdered, regular
	Milk, powdered, skimmed
	Milk, regular
	Milk, skimmed
	Yoghurt, low fat
	Yoghurt, regular
Nuts and seeds (including spices)	Peanut
	Peanut butter
	Sesame seed
RTE savouries	Fish based snack
	Fried chips and cracker
	Popcorn
	Roasted nut snack
Sauces and condiments	Cheese sauce
	Chilli powder
	Chilli sauce
	Cream sauce
	Fish sauce
	Mayonnaise
	MSG
	Mustard sauce
	Oil, sesame
	Oyster sauce
	Pepper, black
	Pepper, white, powder
	Salt
	Sambal chilli / belacan
	Satay sauce
	Soy sauce, dark
	Soy sauce, light
	Sugar, white
	Teriyaki sauce

Food Category	Foods
	Lotus root
	Potato
	Pumpkin
	Sweet potato
	Yam
Stalk, stem and bulb vegetables	Asparagus◊
	Bamboo shoot
	Celery
	Dried lily bulb
	Garlic◊
	Ginger
	Lily bulb◊
	Onion◊
	Spring onion
	Turmeric

◊ Seasonal foods

Food Category	Foods
	Thousand island sauce
	Tomato sauce
	Vinegar
	Wasabi
Vegetable protein	Mock meat (gluten based)
	Mock meat (soy based)

Table S12. Mean concentrations of mycotoxins and phytotoxins, in ppb, for each food category in Singapore's TDS

	Mean concentration [ppb]								
**Food Category**	**Aflatoxin B & G (sum)**	**Aflatoxin M**	**DON & its derivatives**	**Ochratoxin A**	**Fumonisins**	**Zearalenone & its derivatives**	**Ergot Alkaloid**	**Pyrrolizidine Alkaloids**	**Tropane Alkaloids**
**Beverages**	0.13	-	-	-	-	-	-	-	-
**Bakery products**	0.95	-	24.64	0.34	-	-	5.11	-	-
**Fish and seafood**	0.02	-	10.65	-	-	-	-	-	-
**Grains and grain-based products**	0.09	-	13.46	0.15	-	8.07	1.23	-	-
**Meat and meat products**	-	-	-	-	-	-	-	-	-
**Eggs and egg products**	-	-	-	-	-	-	-	-	-
**Fats and oils**	0.22	-	-	-	-	5.99	-	-	-
**Milk and dairy products**	-	-	-	-	-	-	-	-	-
**Fruit and fruit products**	0.02	-	-	-	-	-	-	-	-
**Tap water, drinking water**	-	-	-	-	-	-	-	-	-
**Confectionary**	-	-	-	-	-	10.30	-	5.61	-
**RTE savouries**	0.51	-	-	-	-	-	-	-	-
**Fungi, seaweed**	-	-	-	-	-	-	-	-	-
**Nuts and seeds (including spices)**	0.28	-	-	-	-	-	-	-	-
**Vegetable protein**	-	-	-	0.20	-	-	3.46	-	-
**Brassica vegetables**	-	-	-	-	-	-	-	-	-

	Mean concentration [ppb]								
Food Category	Aflatoxin B & G (sum)	Aflatoxin M	DON & its derivatives	Ochratoxin A	Fumonisins	Zearalenone & its derivatives	Ergot Alkaloid	Pyrrolizidine Alkaloids	Tropane Alkaloids
Fruiting vegetables	-	-	-	-	-	-	-	-	-
Leafy vegetables, herbs	-	-	-	-	-	-	-	-	-
Legumes	1.16	-	-	1.21	-	-	-	-	-
Root and tubers	-	-	-	-	-	-	-	-	-
Stalk, stem and bulb vegetables	-	-	-	12.62	8.81	-	-	-	-
Sauces and condiments	0.48	-	47.20	0.51	65.40	-	-	-	-
Composite foods	-	-	11.30	0.18	-	-	1.87	-	-

Table S13. Mean concentrations of pesticide residues, in ppb, for each food category in Singapore's TDS

	Mean concentration [ppb]		
Food Category	Multi-residues	Dithiocarbamates	ETU/PTU
Beverages (including bottled water)	7.5	-	-
Bakery products	8.6	-	-
Fish and seafood	13.7	-	20.0
Grains and grain-based products	27.3	-	-
Meat and meat products	24.3	-	-

	Mean concentration [ppb]		
**Food Category**	**Multi-residues**	**Dithiocarbamates**	**ETU/PTU**
**Eggs and egg products**	-	-	-
**Fats and oils**	-	-	-
**Milk and dairy products**	-	-	-
**Fruit and fruit products**	39.0	90.7	19.0
**Tap water, drinking water**	-	-	-
**Confectionary**	-	-	-
**RTE savouries**	35.9	-	-
**Fungi, seaweed**	14.4	-	-
**Nuts and seeds (including spices)**	5.6	115.6	-
**Vegetable protein**	5.4	-	-
**Brassica vegetables**	50.0	1,029.4	-
**Fruiting vegetables**	55.2	126.4	26.2
**Leafy vegetables, herbs**	110.6	1,004.0	99.0
**Legumes**	45.5	-	115.5
**Root and tubers**	63.2	-	21.3
**Stalk, stem and bulb vegetables**	103.8	1,957.7	18.6

	Mean concentration [ppb]		
**Food Category**	**Multi-residues**	**Dithiocarbamates**	**ETU/PTU**
**Sauces and condiments**	23.9	-	50.4
**Composite foods**	8.1	-	50.0

Table S14. Mean concentrations of persistent organic pollutants, for each food category in Singapore's TDS

Food Category	Dioxins and dioxin-like PCBs (sum of PCDDs, PCDFs, dioxin-like PCBs) [pg TEQ/g fw]	Perfluorinated compounds [ppb]
**Beverages**	0.01	-
**Bakery products**	0.02	-
**Fish and seafood**	0.10	2.1
**Grains and grain-based products**	0.01	-
**Meat and meat products**	0.03	-
**Eggs and egg products**	0.02	-
**Fats and oils**	0.05	-
**Milk and dairy products**	0.02	-
**Fruit and fruit products**	0.01	-
**Tap water, drinking water**	-	-
**Confectionary**	0.02	-
**RTE savouries**	0.02	-

Food Category	Dioxins and dioxin-like PCBs (sum of PCDDs, PCDFs, dioxin-like PCBs) [pg TEQ/g fw]	Perfluorinated compounds [ppb]
**Fungi, seaweed**	-	-
**Nuts and seeds (including spices)**	0.01	-
**Vegetable protein**	-	-
**Brassica vegetables**	-	-
**Fruiting vegetables**	-	-
**Leafy vegetables, herbs**	-	-
**Legumes**	-	-
**Root and tubers**	-	-
**Stalk, stem and bulb vegetables**	-	-
**Sauces and condiments**	0.02	-
**Composite foods**	0.01	-

Table S15. Mean concentrations of food contact chemicals, in ppm, for each food category in Singapore's TDS

	Mean concentration [ppm]	
**Food Category**	**Bisphenols**	**Plasticisers**
**Beverages**	0.019	-
**Bakery products**	0.002	-
**Fish and seafood**	0.026	0.120
**Grains and grain-based products**	0.017	-
**Meat and meat products**	0.009	0.110
**Eggs and egg products**	-	-
**Fats and oils**	0.009	-
**Milk and dairy products**	0.008	0.170
**Fruit and fruit products**	0.016	0.220
**Tap water, drinking water**	-	-
**Confectionary**	0.012	0.100
**RTE savouries**	0.024	-
**Fungi, seaweed**	0.007	-
**Nuts and seeds (including spices)**	0.003	-
**Vegetable protein**	0.021	-
**Brassica vegetables**	0.003	-
**Fruiting vegetables**	0.004	-

	Mean concentration [ppm]	
**Leafy vegetables, herbs**	0.015	0.110
**Legumes**	0.004	-
**Root and tubers**	-	-
**Stalk, stem and bulb vegetables**	0.006	0.130
**Sauces and condiments**	0.075	-
**Composite foods**	0.011	-
